# Origin and diversification of living cycads: a cautionary tale on the impact of the branching process prior in Bayesian molecular dating

**DOI:** 10.1186/s12862-015-0347-8

**Published:** 2015-04-17

**Authors:** Fabien L Condamine, Nathalie S Nagalingum, Charles R Marshall, Hélène Morlon

**Affiliations:** CNRS, UMR 7641 Centre de Mathématiques Appliquées (École Polytechnique), Route de Saclay, 91128 Palaiseau, France; Department of Biological and Environmental Sciences, University of Gothenburg, Box 461, SE-405 30 Göteborg, Sweden; National Herbarium of New South Wales, Royal Botanic Gardens & Domain Trust, Mrs Macquaries Road, Sydney, NSW 2000 Australia; Department of Integrative Biology and Museum of Paleontology, University of California, 1101 Valley Life Sciences Building, Berkeley, CA 94720-4780 USA; CNRS, UMR 8197 Institut de Biologie de l′École Normale Supérieure, 46 rue d′Ulm, 75005 Paris, France

**Keywords:** Bayesian relaxed-clock, Birth-death process, Branching process prior, Cycadales, Cycadopsida, Speciation tree prior, Yule model

## Abstract

**Background:**

Bayesian relaxed-clock dating has significantly influenced our understanding of the timeline of biotic evolution. This approach requires the use of priors on the branching process, yet little is known about their impact on divergence time estimates. We investigated the effect of branching priors using the iconic cycads. We conducted phylogenetic estimations for 237 cycad species using three genes and two calibration strategies incorporating up to six fossil constraints to *(i)* test the impact of two different branching process priors on age estimates, *(ii)* assess which branching prior better fits the data, *(iii)* investigate branching prior impacts on diversification analyses, and *(iv)* provide insights into the diversification history of cycads.

**Results:**

Using Bayes factors, we compared divergence time estimates and the inferred dynamics of diversification when using Yule versus birth-death priors. Bayes factors were calculated with marginal likelihood estimated with stepping-stone sampling. We found striking differences in age estimates and diversification dynamics depending on prior choice. Dating with the Yule prior suggested that extant cycad genera diversified in the Paleogene and with two diversification rate shifts. In contrast, dating with the birth-death prior yielded Neogene diversifications, and four rate shifts, one for each of the four richest genera. Nonetheless, dating with the two priors provided similar age estimates for the divergence of cycads from *Ginkgo* (Carboniferous) and their crown age (Permian). Of these, Bayes factors clearly supported the birth-death prior.

**Conclusions:**

These results suggest the choice of the branching process prior can have a drastic influence on our understanding of evolutionary radiations. Therefore, all dating analyses must involve a model selection process using Bayes factors to select between a Yule or birth-death prior, in particular on ancient clades with a potential pattern of high extinction. We also provide new insights into the history of cycad diversification because we found *(i)* periods of extinction along the long branches of the genera consistent with fossil data, and *(ii)* high diversification rates within the Miocene genus radiations.

**Electronic supplementary material:**

The online version of this article (doi:10.1186/s12862-015-0347-8) contains supplementary material, which is available to authorized users.

“*Cycads are to the vegetable kingdom what Dinosaurs are to the animal, each representing the culmination in Mesozoic times of the ruling Dynasties in the life of their age*.”

Lester Ward, 1900

## Background

Our understanding of biotic evolution relies heavily on phylogenetic and dating reconstructions that provide insight into the periods of major diversification [1]. In the last decade, the advent of molecular dating approaches has fostered an explosion of studies constructing time-calibrated trees for diverse plant clades like bryophytes [2], ferns [3,4], gymnosperms [5-8] and angiosperms [9-11]. These dated trees have permitted the study of character evolution via the reconstruction of ancestral traits [11], inference of biogeographical history [7], as well as estimates of diversification rates [2,8,10]. Dated trees are thus pivotal to our understanding of the evolution of plants, and of the groups that interact with them, such as herbivores and pollinators [12].

Despite the importance of reliable estimates of divergence times, our understanding of the temporal patterns of diversification remain in flux for many groups, in part because the methods for estimating evolutionary timescales from DNA sequences are being refined [1,13,14]. Since the introduction of relaxed-clock methods, which allow substitution rates to vary across the tree, a range of molecular dating methods has been developed. Bayesian inference has received the most attention because of the flexibility with which different parameters and prior assumptions can be incorporated, and the fact that priors are updated as part of the analysis [15,16]. The use of explicit prior distributions is central to the Bayesian perspective; however, the critical role of prior selection in Bayesian analysis is not always fully appreciated [17].

In Bayesian relaxed-clock (BRC) approaches, there are various types of priors, including priors on calibration points, branch-lengths, clock models, and branching processes. Priors on calibration points have been well studied [18] and, not surprisingly, the choice of these priors can affect estimates of node ages [19]. The effects of branch-length priors on posterior probabilities have been studied; priors assuming long internal branches cause high posterior probabilities [20]. In comparison, the impact of different branching process priors has been relatively under-explored [15,21].

The branching process prior (BPP), also called the ‘tree shape’ or ‘speciation tree’ prior, is a prior model on how trees are generated. Phylogenetic trees are the result of speciation and extinction events, and their relative roles can be varied and represented as different models of diversification [22]. These models effectively place a prior on how phylogenetic trees grow. Probability distributions over models of diversification were employed in some of the earliest attempts to use likelihood techniques to reconstruct genealogies [23]. The two most commonly used BPPs are the Yule (also called ‘pure-birth’) process, which models tree formation with a constant rate of speciation and no extinction, and the birth-death process, which includes speciation as well as a constant rate of lineage extinction. Birth-death priors have been used in Bayesian phylogenetics [24]; however, most published analyses use the Yule prior, perhaps because it was initially the only prior for the diversification process implemented in the widely used Bayesian software package BEAST [16]. Although the birth-death prior has recently been integrated into this software [25,26], many phylogenetic analyses still use the Yule prior (probably because the BEAST manual recommend the use of the Yule prior, see p. 10 of the BEAST manual, version 1.4). Few studies have used both priors in Bayesian dating, and even fewer have compared the impact of prior choice, even though recent studies have started to do so (e.g. [27]). One of the first to use both priors is the study of Couvreur *et al.* [28], who estimated the evolutionary history of the Brassicaceae using a BRC approach with both priors. Their age estimates did not differ under the Yule or birth–death diversification models, and both models fit the data equally well. While these results could be interpreted to mean that the BPP has little influence on BRC reconstruction in general, the Brassicaceae are a relative young group that originated in the Eocene (credibility interval 24.2-49.4 million years ago, Ma) and likely did not experience major extinction events. However, choice of diversification prior might make a substantial difference for groups that have undergone significant extinction, and for which using a pure-birth Yule prior could give in spurious results.

The cycads (Cycadopsida: Cycadales) are a plant group particularly well suited for testing the influence of BPPs on molecular dating. Today’s species (331 species in the tropics and subtropics, [29]) are thought to represent the last remnants of their formidable past, and their evolutionary history extends back to the mid-Permian (~270 Ma) [30-33]. Cycads have witnessed many drastic environmental changes [34-37] and likely suffered major periods of extinction [6,38]. Due to their richness and diversity in Mesozoic fossil records, their current diversity has long been assumed to be of relictual origin dating back to the Late Cretaceous [30,39-42]. This idea has been challenged by molecular time-calibrated phylogenies showing that long branches subtend late Cenozoic (in the Miocene ~15 Ma) and near-simultaneous initiations of diversification of the living genera [6,43].

Despite these recent studies, there remain dating uncertainties in the evolutionary history of cycads. At the generic level, some fossils suggest that the various radiations might be much older than the late Miocene (i.e. Eocene). Indeed, numerous pre-Miocene cycad fossils have been documented (e.g. [44-49]) that are likely early representatives of living genera. For example, recently described fossils from the early Cenozoic of China have been assigned to the crown-group of *Cycas* [50], but that assignment is doubtful given the extensive homoplasy in the characters used to link the fossils to the extant taxon [51]. Even if there is a possibility that fossils instead should be assigned to stem, they cast doubts on the dating. At the level of the whole group, different studies using similar fossil calibrations (but different taxon and molecular sampling) have produced different age estimates, notably for the origin of the cycad crown: ≈200 Ma for Nagalingum *et al.* [6], and ≈ 230 Ma for Salas-Leiva *et al.* [43]. Therefore further dating analyses are needed to estimate the cycad age and validate the recent generic radiations.

In reconstructing the phylogeny of cycads, Nagalingum *et al.* [6] sampled about two-thirds of all known species and used a molecular dataset composed of two chloroplast genes and one nuclear gene (although most of the taxa had the nuclear gene only). In addition to Penalized Likelihood and a strict molecular clock, they inferred the age and divergence times using the BRC approach implemented in BEAST, calibrated with four fossil constraints and a birth-death process as the BPP. The birth-death process used in the Bayesian analyses yielded a high ratio of extinction to speciation (relative death rate, 0.97) [6], suggesting a dominant role of extinction in shaping the phylogeny of cycads. Given our prior knowledge of cycad evolution gleaned from the fossil record, using a prior that includes extinction is realistic. However, Nagalingum *et al.* [6] did not assess the support of the birth-death prior versus other priors.

This study has four objectives: *(i)* investigating the impact of the BPP on the dating of ancient clades using the cycads as an example, *(ii)* assessing whether the birth-death prior is statistically supported, *(iii)* studying the difference between the Yule and birth-death prior on our understanding of cycad diversification, and *(iv)* providing a cycad timetree reconciling fossil and phylogenetic data. We also discuss potential explanations for the differences obtained when using different priors and the consequences of prior choice for Bayesian molecular dating.

## Methods

### Taxon sampling and molecular dataset

**Figure 1:**
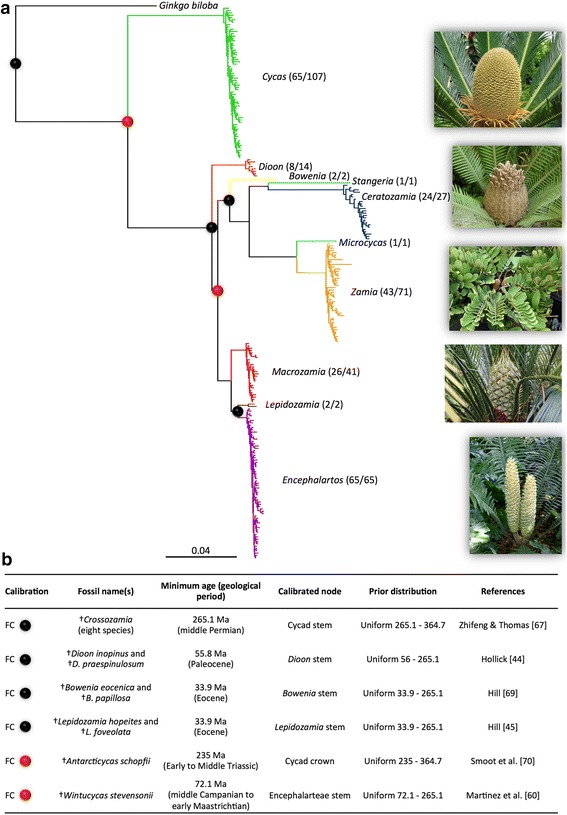
The node calibration procedure used for dating the cycads. **(a)** Phylogenetic tree of cycads showing the relationships among the 10 genera. Genera are represented by triangles proportional to their species richness. Numbers in parenthesis are the number of species sampled, and the total number of species, within each genus. Pictures illustrate *Cycas*, *Dioon*, *Zamia*, *Macrozamia*, and *Encephalartos* species. Black dots indicate the four ‘traditional’ fossil calibrations used for dating, and red dots indicate the two new fossil calibrations evaluated in this study. **(b)** Information related to the four fossil calibrations (FC, see the text for more details). Ma, million years ago.

We extended the molecular dataset of Nagalingum *et al.* [6] that initially contained 199 cycad species. We added molecular data for 38 additional species and the same genes retrieved from Genbank [52-57]. Our dataset comprises three genes covering two plastid genes: the maturase K (matK, 2,387 nucleotides, 82 taxa) and the ribulose 1,5-bisphosphate carboxylase large subunit (rbcL, 1,398 nucleotides, 80 taxa), and one nuclear gene: region 1 of phytochrome (PHYP, 1,802 nucleotides for 201 taxa). This resulted in a total of 237 species out of 331 described species (71%), representing all extant genera with the following number of species per genus: 2 of the 2 *Bowenia* (*B. serrulata* and *B. spectabilis*), 65 of the 107 *Cycas*, 24 out of 27 *Ceratozamia*, 8 of the 14 *Dioon*, all of the 65 *Encephalartos*, 2 of the 2 *Lepidozamia* (*L. hopei* and *L. peroffskyana*), 26 of the 41 *Macrozamia*, the monotypic *Microcyas calocoma*, the single *Stangeria eriopus*, and 43 out of 71 *Zamia* (including the junior synonym genus *Chigua*) (Figure 1). Following Nagalingum *et al.* [6], the dataset also included six species as outgroups: *Ginkgo biloba* (Ginkgoales), which is recognised as the sister lineage of cycads [58]; plus five conifer species (*Abies firma*, *Araucaria heterophylla*, *Cryptomeria japonica*, *Pinus strobus* and *Pseudotsuga menziezii*). The phylogenetic analyses were thus performed on a dataset containing a total of 243 species (of which 237 are cycads) and 5587 nucleotides. The information on each sampled species is presented on Additional file 1: Table S1.

### Fossil calibrations

To calibrate the cycad tree, we used two fossil datasets. First, we followed Nagalingum *et al.* [6] and used four cycad fossil constraints (FC1-4), which were retrieved from a careful examination of the cycad fossil record ([32]; Figure 1). These four fossils have also been used by Salas-Leiva *et al.* [43]. Hereafter this fossil dataset is referred to as the ‘traditional fossil dataset’ (FC1-4). Second we used these four fossils and added two fossils based on the phylogenetic analyses of Hermsen *et al.* [32] and Martínez *et al.* [59]. The fossil record was re-examined after new fossils from the Cretaceous were discovered, which did not affect overall fossil assignments, but allowed the use of new fossils to calibrate the cycad tree [59], two of which are tentatively used here. Hereafter this fossil dataset is referred to as ‘new fossil dataset’ (FC1-6).

Based on the presence of synapomorphies linking the fossils to the extant clades, all fossils were used as minimum age constraints for specific nodes ([32,59], Figure 1). However, since synapomorphies can evolve anywhere along the stem branch and the fossil may attach anywhere along this branch [60], we used a commonly employed approach whereby the fossils constrain the stem rather than crown node [61,62]. The absolute fossil ages we used (detailed below) are slightly different from those used in Nagalingum *et al.* [6], mainly due to an updated geological timescale [63]. Dating fossils older than ~50,000 years is difficult, which means that those fossils are typically assigned to a stratigraphic interval, for example, the late Miocene. The ages assigned below are the ages of the youngest of the boundaries of stratigraphic interval within which the fossil was found (the fossil will actually be older than this age designation).

#### Cycad stem (FC1, Younger = 265.1 Ma and Older = 364.7 Ma)

The oldest possible records of the group Cycadophyta are microstrobili (e.g. [64]) and megasporophylls (e.g. [65,66]), of which †*Crossozamia* (eight known species) is the least equivocal in terms of phylogenetic affinity [32,59,66]. *Crossozamia* consists of megasporophylls with similar morphology to the extant *Cycas* [65,66]. Although Hermsen *et al.* [32] identified *Crossozamia* as being sister to *Cycas*, the two characters supporting this relationship do not provide strong evidence for *Crossozamia* belonging to crown group cycads [59]. In addition, the loosely aggregated cone is most probably an ancestral state within cycads. Therefore the divergence between cycads and *Ginkgo biloba* was constrained using *Crossozamia*. The age for the host rock formation (Shihhotse) that preserves the *Crossozamia* fossils was initially described as lower Permian, but the age of the Shihhotse Formation has been revised to the Roadian-Wordian (265.1-272.3; [63]) in the middle Permian [67]. We used the minimum age of this time interval (265.1 Ma) as a minimum age for the stem of cycads. This age was also used as a conservative maximum possible age for the nodes with internal fossil calibrations (FC2-FC4).

#### Dioon *stem (FC2, Younger = 56 Ma and Older = 265.1 Ma)*

The origin of the stem group of *Dioon* was constrained by fossil leaves of †*D. inopinus* and †*D. praespinulosum* [44], which have synapomorphies consistent with the leaflet venation anastomoses and leaflet insertion on the rachis of extant *Dioon* [32]. While originally described as Eocene by Hollick [44], a more recent interpretation of the composition of the Hamilton Bay flora (Kootznahoo Formation), Kupreanof Island, Alaska, from which fossils of both *Dioon* were originally described, suggest they are of Paleocene age [6,32]. Therefore the *Dioon* stem was assigned a minimum age of 56 Ma [63].

#### Bowenia *stem (FC3, Younger = 33.9 Ma and Older = 265.1 Ma)*

*Bowenia* leaf fossils of †*B. eocenica* and †*B. papillosa* [68], both found in Australia, were used to constrain the divergence of *Bowenia* from other genera. These fossils were identified as members of the extant genus based on cuticular characters (i.e. number of subsidiary cells) and leaflet morphology (i.e. venation and serrated margin). Both fossil species were found in Australian Eocene deposits (at Anglesea, Victoria for †*B. eocenica*, and at Nerriga, New South Wales for †*B. papillosa*), which confers a minimum age constraint at 33.9 Ma for the *Bowenia* stem [63].

#### Lepidozamia *stem (FC4, Younger = 33.9 Ma and Older = 265.1 Ma)*

Fossil *Lepidozamia* leaves of †*L. hopeites* and †*L. foveolata* are also from Australia [45]. Fossils of this genus can be identified by cuticular characters (orientation of the epidermal cells relative to axis of the pinna) that are unique to *Lepidozamia* and support affinity to the extant genus [32,45]. Found in the Australian Eocene (at Nerriga, New South Wales for †*L. foveolata*), this set a minimum age of 33.9 Ma for the *Lepidozamia* stem [63].

#### Cycad crown (FC5, Younger = 235 Ma and Older = 364.7 Ma)

The age of modern cycads can be calibrated with †*Antarcticycas schopfii* [69]. The fossil *Antarcticycas* has been extensively studied [32,70]. A reconstruction of the plant habit has even been proposed [70]. This makes *Antarcticycas* the most completely known of the extinct Triassic cycad taxa, if not of all fossil cycads, due to the presence of anatomically preserved organs [70]. Interestingly, phylogenetic studies have also tentatively assigned *Antarcticycas* within modern cycads, and close to the crown of cycads [32,59]. None of the extant genera are phylogenetically sister to *Antarcticycas*, which makes it a valuable fossil calibration for the crown of cycads. *Antarcticycas* was found in the Fremouw Formation of the early Middle Triassic of Antarctica. We used a conservative age of the Middle Triassic, i.e. 235 Ma [63], although there remains uncertainty on the true age of the Fremouw Formation with some authors suggesting an Anisian age (242-247.2 Ma).

#### Encephalarteae *stem (FC6, Younger = 72.1 Ma and Older = 265.1 Ma)*

Many fossils have been attributed to the tribe Encephalarteae based on morphological and phylogenetic evidence [32,59]. Among them the recently discovered *†Wintucycas stevensonii* [59] is one of the best-known fossils. *Wintucycas* has features that clearly allow us to assign it to Encephalarteae due to their manoxylic wood, centripetal polyxyly, parenchymatous pith, centrifugal polyxyly and medullary vascular bundles. *Wintucycas* was found in the Allen Formation (middle Campanian to early Maastrichtian) of the Late Cretaceous of Argentina (Patagonia), providing a minimum age for the stem of Encephalarteae at 72.1 Ma [63].

#### Tree root height

The tree root height is the divergence between Cycadales and Ginkgoales. Following Clarke *et al.* [62], a maximum age can be established with the first records of seeds in the form of preovules that satisfy the criteria of the seed habit. These criteria are the possession of a single functional megaspore that is enveloped in a nucellus (considered equivalent to the megasporangium), which is surrounded (to some extent) by an integument or pre-integument and has mechanisms enabling the capture of pollen before seed dispersal [62,71]. All the criteria are first met with †*Elkinsia polymorpha* found in the VCo spore Biozone, Evieux Formation [71] in the Fammenian (Late Devonian). The VCo spore Biozone spans 364.7-360.7 Ma [63]. A maximum age for gymnosperms is thus 364.7 Ma. We also used this age to set maximum ages for the cycad stem (FC1) and crown (FC5).

### Phylogenetic reconstructions

To obtain a starting tree for the dating analyses, Bayesian inferences were performed with MrBayes 3.2.3 [72]. To determine the best-fit partitioning scheme of molecular evolution for our dataset, we used PartitionFinder 1.1.1 [73]. For the PartitionFinder analyses, branch lengths were *unlinked* to allow them to be independently estimated for each partition. The searched for best model, among those available in MrBayes, was performed under the *greedy* algorithm based on the Bayesian Information Criterion model metric. We used the partitioning scheme and among-site rate variation suggested by PartitionFinder, but instead of selecting one substitution model *a priori*, we used reversible-jump Markov Chain Monte Carlo (rj-MCMC) to allow sampling across the entire substitution rate model space [74].

MrBayes analyses consisted of four rj-MCMC running for 100 million generations with sampling every 10,000 generations and the first 25% discarded as burn-in. We specified *(i)* a uniform prior probability of phylogenies (i.e. all possible trees are considered *a priori* equally probable), and *(ii)* a uniform prior probability distribution on branch lengths. The convergence of the runs was assessed by checking the potential scale reduction factor (PSRF) values of each parameter in MrBayes and the Effective Sample Size (ESS) values of each parameter in Tracer 1.6 [75]. Values of PSRF close to 1.00 and ESS above 200 were considered as good indicator of convergence. Nodes recovered with posterior probabilities (PP) ≥ 0.95 are considered to be strongly supported.

### Bayesian relaxed-clock analyses

For all dating analyses, we used the BRC approach as implemented in BEAST 1.8 [26]. BEAUti 1.8 was used to create the BEAST input file. We implemented partitioned relaxed-clock models with an uncorrelated lognormal clock model that assumes an underlying lognormal distribution (UCLD) of the evolutionary rates [16], which is more likely to yield accurate estimates than the uncorrelated relaxed clock model that assumes an exponential distribution of the evolutionary rates [76]. We used the same nucleotide substitution model as for the MrBayes analyses. Given that one of the main goals of the study was to investigate the impact of the BPP on molecular dating, we set the tree prior in BEAST to either the Yule or the birth-death process [23]. Following the rigorous approach of Vanneste *et al.* [77], we utilized the following priors: a uniform prior between 0 and 10 with a starting value at 0.1 for the Yule birth rate and birth-death mean growth rate, and a uniform prior between 0 and 1 with a starting value at 0.5 for the birth-death relative death rate. We used an exponential prior with mean one-third on the standard deviation of the UCLD model, and a uniform prior between 0 and 1 on the mean of the UCLD model. Fossil calibrations were conservatively set with a uniform distribution bounded by the minimum and maximum ages as explained above. A starting tree with branch lengths satisfying all of the fossil prior constraints was inserted, as derived from the MrBayes analyses (described above) [77]. The ‘tree operators’ of the BEAST tree model were disabled to keep the topology fixed so that only the branch lengths were optimized. This procedure ensures better convergence of the tree topology (S. Ho, pers. comm.). Other parameters were kept to their default prior distribution or were indirectly specified through other parameters.

We designed four distinct BEAST analyses by mixing the fossil dataset and the BPP as follows *(i)* use of the ‘traditional fossil dataset’ and the Yule model as BPP; *(ii)* use of the ‘traditional fossil dataset’ and the birth-death process as BPP; *(iii)* use of the ‘new fossil dataset’ and the Yule model as BPP; and *(iv)* use of the ‘new fossil dataset’ and the birth-death process as BPP. For each fossil dataset, we included all fossils for dating the tree, and did not perform cross-validation analyses because Warnock *et al.* [78] questioned the cross-validation approach to measure consistency among calibrations based on minimum constraints [61,79]. The most effective means of establishing the quality of fossil-based calibrations is through *a priori* evaluation of the intrinsic palaeontological, stratigraphic, geochronological and phylogenetic data [78], which we describe above.

The MCMC analyses were run for 100 million generations and sampled every 10,000 generations, resulting in 10,000 trees in the posterior distribution; we discarded the first 2,500 trees as burn-in. We used BEAGLE [80], a library for high-performance statistical phylogenetic inference, with default parameters. This allowed a faster and more accurate computation of likelihood models within our BEAST analyses. All BEAST analyses were performed on the computer cluster CIPRES Science Gateway 3.3 ([81]; http://www.phylo.org/). Tracer was used to assess graphically the convergence of runs, and to check the ESS for all parameters (indicated by ESS above 200). For each analysis, we conducted two independent runs to ensure convergence of the MCMC. Post burn-in trees from the two distinct runs (7,500 trees for each run) were further combined to build the maximum clade credibility tree.

In Bayesian analyses, models have traditionally been compared using the harmonic mean estimate (HME). Other approaches have been developed to calculate accurately the marginal likelihood estimate (MLE) of the specified model, such as path sampling (PS) [82] and stepping-stone sampling (SS) [83]. PS and SS substantially outperform the HME estimator, which overestimates the marginal likelihood and fails to select reliably the best model [76]. We performed MLE using SS with 150 path steps, each with a chain length of one million iterations (G. Baele, pers. comm.), and other parameters were set by default. We directly calculated the log-Bayes factors (hereafter BF) [76,84] from MLEs, and used BF to compare the support of the Yule and the birth-death processes for a given calibration strategy. We considered BF values above 5 to indicate that one model was significantly favoured over another [84].

### Macroevolutionary rates through time

We used the Bayesian Analysis of Macroevolutionary Mixture (BAMM, www.bamm-project.org) to estimate speciation and extinction rates through time and among/within clades [85-87]. BAMM is an analytical tool for studying complex evolutionary processes on phylogenetic trees, potentially shaped by a heterogeneous mixture of distinct evolutionary dynamics of speciation and extinction across clades. The method uses rj-MCMC to detect automatically rate shifts and sample distinct evolutionary dynamics that best explain the whole diversification dynamics of the clade. Within a given regime, evolutionary dynamics can involve time-variable diversification rates; in BAMM, the speciation rate is allowed to vary exponentially through time while extinction is maintained constant [86]. Subclades in the tree may diversify faster (or slower) than others, and BAMM detects these diversification rate shifts without *a priori* hypotheses on how many and where these shifts might occur. BAMM provides estimates of marginal probability of speciation and extinction rates at any point in time along any branch of the tree. Marginal probabilities of the number of evolutionary regimes can also be computed, allowing comparisons of models with a given number of shifts with BF.

Estimating extinction rates from reconstructed phylogenies is notoriously difficult [88-90], although not impossible (see Box 3 of Morlon [22] for a discussion of the subject, and ref. [91-94] for recent papers contradicting the widespread idea that extinction rates cannot be estimated from molecular phylogenies). The developer of the approach cautions that BAMM estimates of extinction should be taken with care [87], because they are sensitive to the choice of the prior and have large confidence intervals. Still, these estimates are well correlated with the underlying rates (Figure five B in Rabosky [87]). Biases in extinction rate estimates often come from fitting models based on underlying hypotheses that are violated in nature, such as fitting models with homogeneous rates across clades when major rate shifts occurred (see ref. [90,93], but see [94]). The BAMM approach should in principle help with these issues, because it explicitly accounts for rate heterogeneity across clades while allowing rates to vary over time [85-87]. We share Beaulieu and O’Meara’s view [94] that we should use prudent caution, but not abandon all hope, of estimating extinction from molecular phylogenies. Hence, we think that it is crucially important to assess the effect of BPP prior choice on these estimates, and to discuss them in the light of what is known from the cycads fossil record.

BAMM is implemented in a C++ command line program and the BAMMtools R-package [87]. We ran BAMM analyses on the timetree calibrated with the ‘new-fossils dataset’, and reconstructed with either the Yule or the birth-death prior. We set four rj-MCMC running for 50 million generations and sampled every 50,000 generations. A compound Poisson process is implemented in BAMM for the prior probability of a rate shift along any branch. We used a prior value of 1.0 implying a null hypothesis of no rate shift across the phylogeny, as recommended by Rabosky [87]. We accounted for incomplete taxon sampling using the implemented analytical correction, with a sampling fraction set to 0.716 (i.e. 237 species out of the 331 described species). We performed four independent runs (with a burn-in of 10%) using different seeds, and we used ESS to assess the convergence of the runs, considering values above 200 as indicating convergence. The posterior distribution was used to estimate the configuration of the diversification rate shifts; alternative diversification models were compared using BF. To complement these analyses, we computed pairwise probabilities that any two lineages or clades share a common set of macroevolutionary rate parameters, using the macroevolutionary cohort analysis implemented in BAMM [87].

## Results

### Phylogeny of cycads

Tracer plots indicated that MrBayes runs reached convergence before 10 million generations (results not shown). Convergence of the analyses was also supported by the average standard deviation of split frequencies (<0.01), all PSRF values close to 1.0 (range between 0.999 and 1.019), and ESS values above 200 (with many >1000) for the post burn-in trees. The resulting Bayesian trees were well resolved: all nodes of the backbone tree and genus crown nodes were recovered with maximal posterior probabilities (PP = 1, Figure 1 and Additional file 2: Figure S1). Only nodes within genus radiations were recovered with lower PP, which was expected because the genetic divergence among cycad species within genera is low (*Cycas*: [95,96]; *Ceratozamia*: [52]; *Dioon*: [56]; *Encephalartos*: [97]; *Macrozamia*: [98]; *Zamia*: [99]). Our MrBayes analyses are congruent with the results of Nagalingum *et al.* [6], but differ from other studies [43,54,55,100]. Notably the genus *Bowenia* is found as sister to the clade ((*Ceratozamia*, *Stangeria*), (*Microcycas*, *Zamia*)) as in Nagalingum *et al.* [6], but an alternative position is that *Bowenia* is sister to all genera, except *Cycas* and *Dioon* [43].

### Divergence time estimates and model selection for the branching process prior

**Figure 2:**
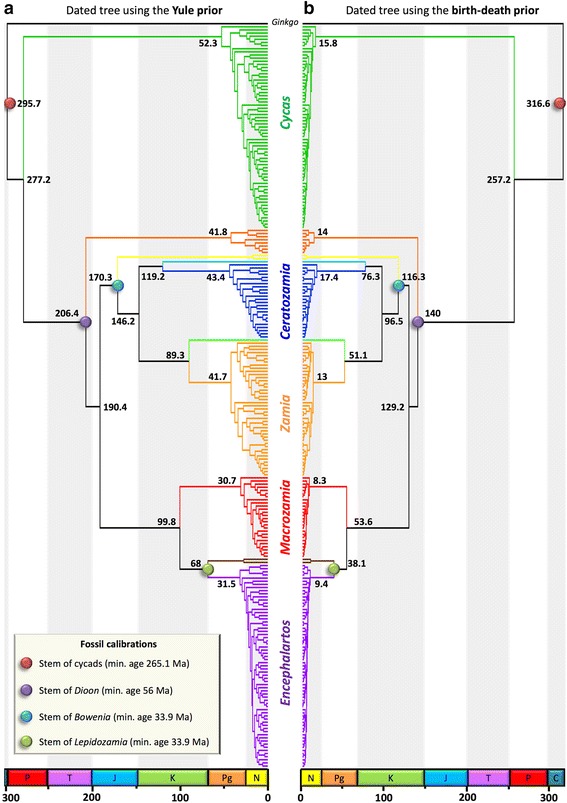
Time-calibrated phylogeny of Cycadales obtained with the four fossil calibrations. Timetree obtained with the Yule **(a)** or the birth-death **(b)** model as branching process prior. Each tree is the maximum clade credibility tree with median ages from the Bayesian analyses. The coloured dots highlight nodes on which fossil age constraints were applied. Values are median age estimates for the main nodes, in million years. C, Carboniferous; P, Permian; T, Triassic; J, Jurassic; K, Cretaceous; Pg, Paleogene; N, Neogene. The last geological period, the Quaternary, is missing.

**Figure 3:**
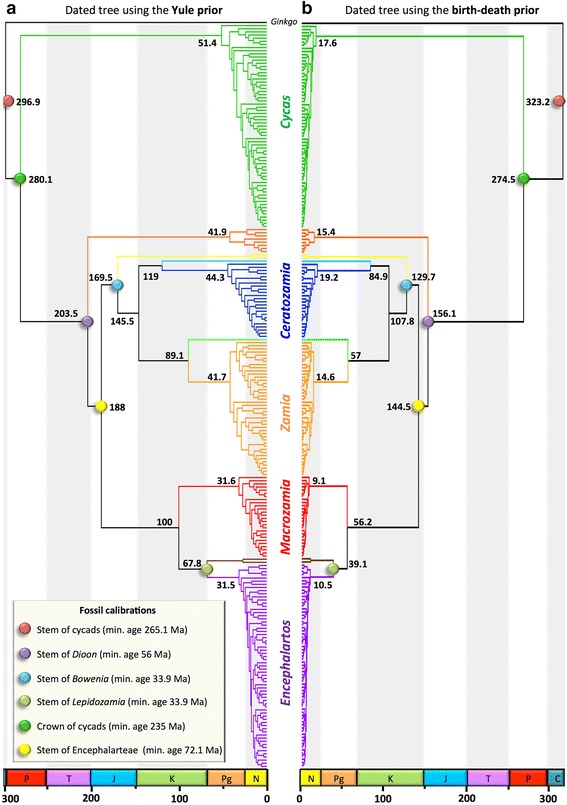
Time-calibrated phylogeny of Cycadales obtained with the six fossil calibrations. Timetree obtained with Yule **(a)** or the birth-death **(b)** model as branching process prior. Each tree is the maximum clade credibility tree with median ages from the Bayesian analyses. The coloured dots highlight nodes on which fossil age constraints were applied. Values are median age estimates for the main nodes, in million years. C, Carboniferous; P, Permian; T, Triassic; J, Jurassic; K, Cretaceous; Pg, Paleogene; N, Neogene. The last geological period, the Quaternary, is missing.

**Figure 4:**
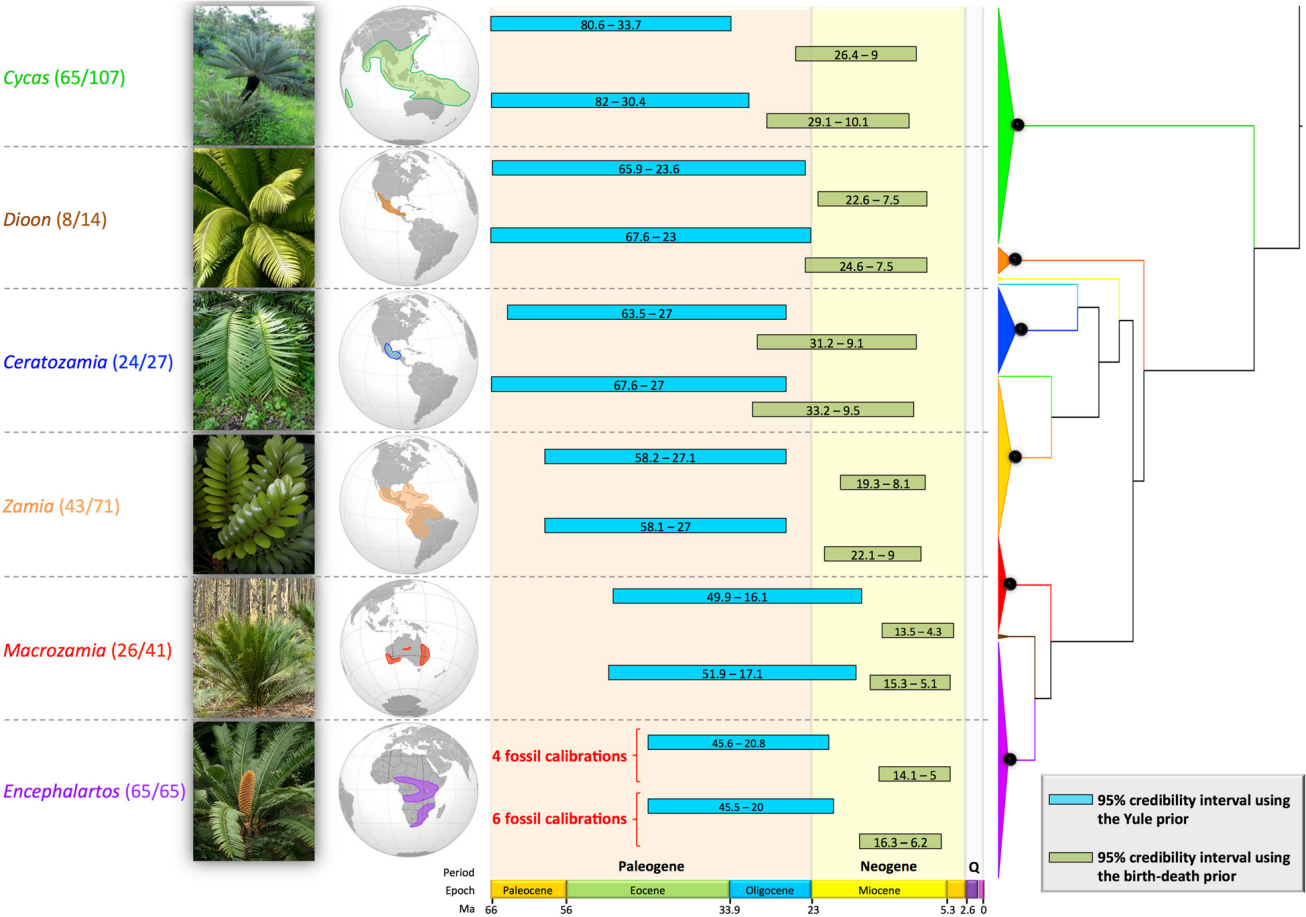
Credibility intervals (95% highest posterior density) for the crown ages of the six most species-rich genera. The blue bars depict the age estimates when using the Yule prior, and the green ones show the age estimates obtained with the birth-death prior, shown for the five dating analyses using most fossil calibration points. Analyses with the Yule prior indicate a Paleogene origin, while analyses with the birth-death prior indicate a Neogene origin. Absolute ages are in million years. Q, Quaternary.

**Figure 5:**
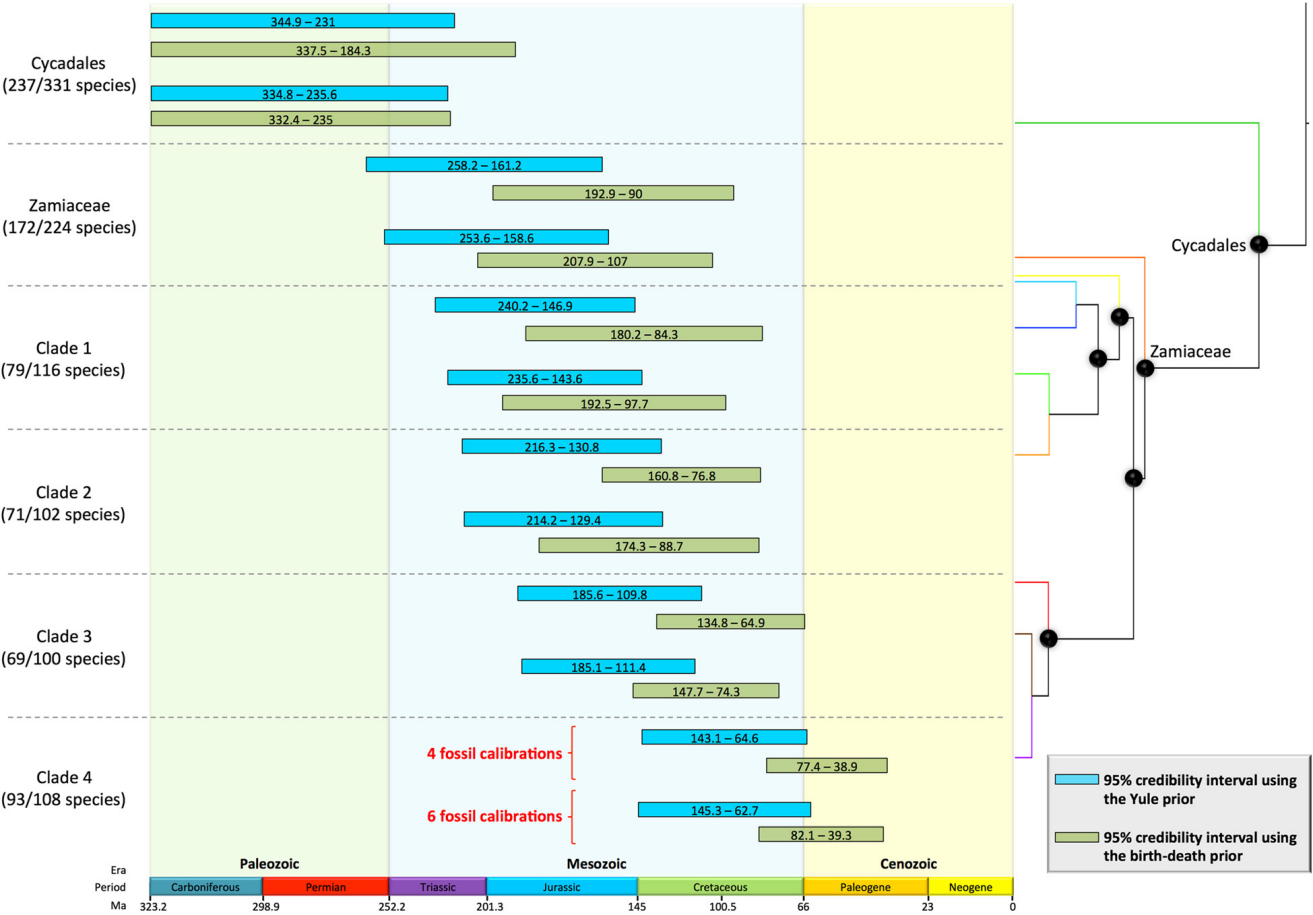
Credibility intervals (95% highest posterior density) for the ages of the six deepest nodes. The blue bars depict the age estimates when using the Yule prior, and the green ones show the age estimates obtained with the birth-death prior, shown for the five dating analyses using most fossil calibration points. The numbers associated to each clade correspond to the numbers on the phylogeny. Analyses with the Yule prior consistently indicate older ages. Absolute ages are in million years. The last geological period, the Quaternary, is missing.

Convergence of the dating analyses was ensured by ESS values above 200 for all parameters (with many >1000) for the post burn-in trees (Table 1) and Tracer plots indicated that BEAST runs reached convergence before the burn-in threshold. The choice of branching process prior had a striking effect on age estimates (Figures 2 and 3; Table 1). Analyses with the Yule prior inferred much older ages for cycad genera (Figure 4). Genus-level crown ages were on average three times older with a Yule than with a birth-death prior (3.2-fold difference with the ‘traditional fossil dataset’ and 2.9-fold with the ‘new fossil dataset’). This difference was quite striking, with non-overlapping credibility intervals (95% highest posterior density) for the genus crown ages (Figure 4). Generic stem ages and some ages of deeper nodes were also inferred to be older with the Yule than with the birth-death prior, although the difference was not as marked as in the case of the genus crown ages (Figure 5). However, the birth-death and Yule priors usually inferred similar ages for the cycad stem and crown ages (Figure 5). Analyses with the birth-death process inferred a very high estimate of the relative extinction rate (ratio of extinction to speciation, or turnover) with a median = 0.966 for the dating with four FC (95% HPD 0.9255-0.9972), and a median = 0.962 for the dating with six FC (95% HPD 0.9153-0.9947).

**Table 1:** **Age estimates for the six nodes subtending a fossil calibration (FC)**

**Fossil calibration strategy**	**Prior**	**HME**	**MLE (SS)**	**ESS (logL)**	**BF**	**Cycad stem (FC1)**	***Dioon*** **stem (FC2)**	***Bowenia*** **stem (FC3)**	***Lepidozamia*** **stem (FC4)**	**Cycad crown (FC5)**	**Encephalarteae stem (FC6)**
						**min. age 265.1 Ma**	**min. age 56 Ma**	**min. age 33.9 Ma**	**min. age 33.9 Ma**	**min. age 235 Ma**	**min. age 72.1 Ma**
Traditional fossil dataset	Birth-death	−26343.61	−26828.83	2451	26.7	316.6 [269.5-363.3]	140 [90-192.9]	116.3 [76.7-160.8]	38.1 [33.9-50.9]	257.2 [184.3-337.5]	129.2 [84.3-180.2]
	Yule	−26330.62	−26855.49	3835		295.7 [265.1-350.1]	206.4 [161.2-258.2]	170.3 [130.8-216.3]	68 [41.7-100.4]	277.2 [231-344.9]	190.4 [146.9-240.2]
New fossil dataset	Birth-death	−26343.36	−26825.04	2076	36.3	323.2 [273.9-364.6]	156.1 [107-207.9]	129.7 [88.7-174.3]	39.1 [33.9-55]	274.5 [235-332.4]	144.5 [97.7-192.5]
	Yule	−26331.16	−26861.34	1361		296.9 [265.1-351.7]	203.5 [158.6-253.6]	169.5 [129.4-214.2]	67.8 [40.6-102]	280.1 [235.6-334.8]	188 [143.6-235.6]

Absolute ages and their credibility intervals were obtained for the two pairs of Bayesian calibration strategies, using either the Yule or the birth-death model as branching process prior.

Values are the median ages and 95% Highest Posterior Density (HPD). We denote HME: harmonic mean estimate; MLE (SS): marginal likelihood, estimated with the stepping-stone sampling; ESS: effective sample size; BF: Bayes factor (BF), computed as the difference between the MLE of the birth-death and the Yule prior. A positive value indicates support for the birth-death prior.

Bayes factor values, calculated with the marginal likelihood estimates of the stepping-stone analyses, support the birth-death process as the best tree prior (Table 1). Of the two pairs of BEAST analyses (four FC and six FC), BF values are above the standard threshold: BF_4FC_ = 26.66 between the Yule and birth-death prior, and BF_6FC_ = 36.3 between the Yule and birth-death prior. On the contrary, the HME found the opposite, that is the Yule model is the best prior for both datasets for all analyses (Table 1). The results for the cycads thus support previous results showing that the HME estimator is not reliable [76].

### Macroevolutionary rates through time

**Figure 6:**
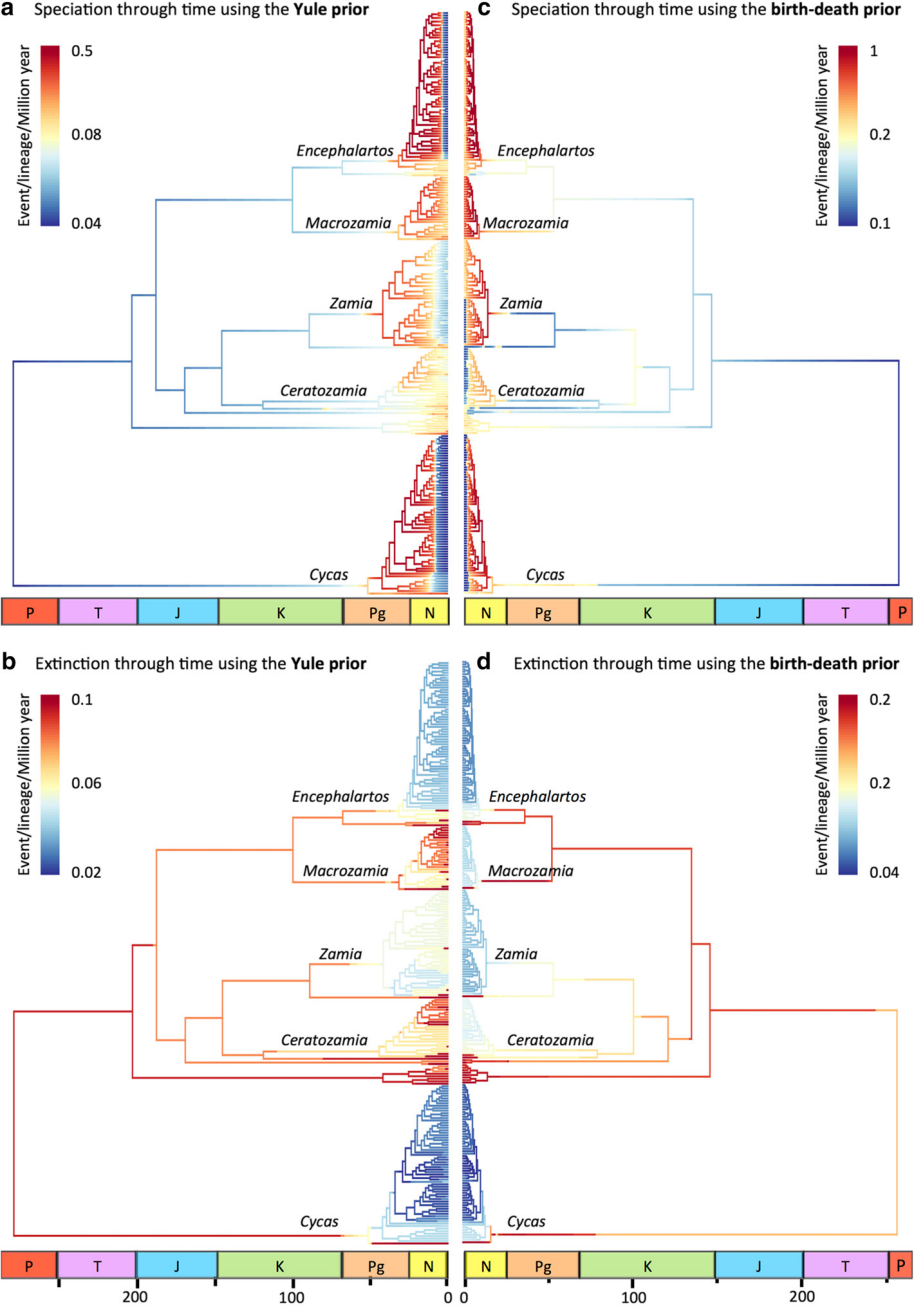
Diversification pattern of Cycadales. Estimates of speciation **(a**,**c)** and extinction **(b**,**d)** rates along the cycad phylogeny obtained from BAMM analyses, when considering the chronogram reconstructed with the Yule **(a**,**b)** or the birth-death **(c**,**d)** prior. Colours at each point in time along branches denote instantaneous rates of speciation or extinction inferred as the mean scenario, with colours indicating mean rates across all the shift configurations sampled in the Bayesian posterior. The diversification scenarios obtained with one versus the other prior are strikingly different. Note the differences for the estimated speciation and extinction rates with each tree prior (rates are twice higher with the tree constrained with a birth-death process). P, Permian; T, Triassic; J, Jurassic; K, Cretaceous; Pg, Paleogene; N, Neogene. The last geological period, the Quaternary, is missing.

The BAMM analyses converged for both trees (ESS_Yule_ = 719.6, Additional file 3: Figure S2; ESS_birth-death_ = 750.6, Additional file 4: Figure S3). The choice of the BPP had a major influence on the number of different evolutionary regimes detected in the history of the cycads, as well as on the estimation of speciation and extinction rates across the tree (Figure 6). Analyses with the Yule prior supported a model with three evolutionary regimes (i.e. two rate shifts, Additional file 5: Figure S4) located at the crown of the genera *Cycas* and *Encephalartos* (according to BF values > 10, and BF = 154.5 over the null model). On the other hand, analyses with the birth-death prior supported a model with five evolutionary regimes (i.e. four rate shifts, Additional file 6: Figure S5) located at (or near) the crown of the four most species-rich genera (according to BF values > 10, and BF = 29.3 over the null model). Moreover, both speciation and extinction rates estimated with the birth-death prior are twice as high as those estimated with the Yule prior (see scales on Figure 6). The credible set of shift configurations with the highest posterior probabilities is provided in Additional file 7: Figure S6 for each tree. The best configuration shift is provided in Additional file 8: Figure S7 for each tree. The macroevolutionary cohort analyses showed distinct evolutionary trajectories for the four richest cycad genera with the birth-death prior (Additional file 9: Figure S8), but not with the Yule prior (Additional file 10: Figure S9).

## Discussion

### The branching process prior impacts Bayesian divergence time estimates

Our Bayesian dating analyses highlight an important effect of the branching process prior on the divergence times of cycads. Dating with the Yule prior consistently inferred ages for genus crown groups that were about three times older than those obtained with the birth-death prior. The cycad tree dated with the birth-death process suggested the initiation of crown radiations of the species-rich genera in the Neogene, whereas the Yule model indicated that the radiations began in the Paleogene. These differences in crown ages are noteworthy because the 95% credibility intervals for these nodes did not overlap. It is important to note that the effect of BPP is reduced towards the origin of cycads since, for both priors, genus radiations are subtended by long stem branches originating in the Triassic or Permian. This pattern is recovered for both calibration strategies. Therefore, the BPP is an important influence on the Bayesian dating analyses.

### Potential causes of age differences between branching process priors

It is unclear why differing tree priors have a major impact on divergence time estimates. The priors differ principally in the inclusion of an extinction rate. In the Yule prior, there is no extinction, but in the birth-death prior there is a parameter for the extinction rate. The effect of extinction in the birth-death prior is that there are relatively greater nodes toward the present compared to the past, because extinction has not yet had an effect on the more recent nodes, this phenomenon is known as the “pull of the present” [25]. This pattern could explain the contrasting results found when dating the cycad phylogeny with a birth-death versus a Yule prior. Support for the birth-death prior includes fossil evidence indicating that extinctions occurred and were important in shaping cycad evolution. More generally, extinction is a dominant feature of life given that 99.9% of all species that ever existed are now extinct [101]. But the role and effect of extinction could also potentially explain why the effect of BPPs appears to be taxon specific (i.e. little effect in Brassicaceae [28]). Indeed, it is possible that older clades might be more sensitive to the BPP choice because extinction is likely to be a more important component of their evolutionary histories. Earlier simulations and empirical studies have indicated that our understanding of the statistical properties of the diversification prior combined with prior distribution of calibrations is incomplete, particularly when the tree topology is considered [78,102,103]. It is an open question as to how important BPP choice will be for other groups, but further analyses of additional taxa as well as simulation studies will be valuable in understanding the detailed effects that tree priors have on age estimates.

### Other potential biases in the branching process priors

While accounting for extinction in BPP priors is an important first step, a birth-death model with homogeneous rates across time and across the various clades within large groups is still an oversimplified model of their evolution. In particular, the hypothesis of rate homogeneity is thought to strongly bias extinction rate estimates [90,93]. We suggest that more complex priors should be developed for implementation in dating software; indeed evidence for age- or time-dependent diversification in numerous clades have resulted in development of methods to take into account rate-heterogeneity through time and clades [93,104].

Another potential bias in BPP priors is the use of models assuming complete or uniform sampling. Phylogenies are often sparsely sampled, particularly in long-branched outgroups. Sparsely sampled outgroups violate the basic assumption of most (if not all) the tree priors implemented in Bayesian dating, namely that the taxon sampling is random and consistent across all clades in the tree (see p. 98 of Drummond and Bouckaert [105]). In this case, it is difficult to find appropriate priors describing the tree: neither the single-parameter Yule model nor the two-parameter birth-death model fit a situation in which some parts of the tree are densely sampled, while others consist of a single long-branched species (i.e. *Ginkgo* versus the cycads).

### Implications for Bayesian dating analyses

The vast majority of molecular dating studies using BEAST have relied on the Yule model, although some recent studies have used both the Yule and birth-death process [27,28,106]. However these studies did not find any differences in age estimates between the two models, nor they did not perform MLE and Bayes factors analyses. Here we tested competing evolutionary hypotheses using a Bayesian relaxed-clock under two different branching priors, and we found stronger support for the birth-death than the Yule process as a prior to reconstruct the cycad tree.

Model selection is a necessary step, because as in the case of cycads, prior choice can have important implications if one simply reconstructs the tree using a Yule model as commonly performed. Conclusions on the age, diversification and/or biogeographic patterns might be different regarding the BPP (see below). Supposedly ancient plant clades might be affected by prior choice as for cycads; specifically, clades with an old and well-documented fossil record that probably experienced periods of extinction such as conifers [7,8], ferns [3,4], gnetophytes [5], and early diverging angiosperm lineages [107,108]. One solution might be to replace the commonly used Yule model by the birth-death process, which is more realistic since all clades are likely to have experienced extinction, even young clades currently diversifying.

As a cautionary tale, we suggest that users routinely test between priors to select the prior that best fit their data. Estimators of the marginal likelihood [82,83] are included in BEAST; therefore, model selection can be relatively straightforward. Furthermore, it has been demonstrated that these marginal likelihood estimators accurately compute BF for BRC studies [76], indicating that the results of our empirical study showing the impact of the BPP are reliable.

### A late Permian origin for cycads

According to the dating scenario supported by BF (i.e. timetree reconstructed with six fossil calibrations and a birth-death process), cycads and *Ginkgo* diverged in the late Carboniferous – early Permian, and the most recent common ancestor of the living cycads is from the late Permian (274.5 Ma). Compared to other dating studies, our timetree fits well with recent works on the genus ages and radiations, but our tree pushes back the origin of cycads more than 50 million years earlier (≈200 Ma for Nagalingum *et al.* [6]; ≈230 Ma for Salas-Leiva *et al.* [43]). For the latter, our results are in agreement with other dating studies that used age constraints on the cycad crown (≈280 Ma for Won & Renner [5]; ≈270 Ma for Burleigh *et al.* [109]). Our cycad timeframe is also congruent with the cycad insect pollinator of the order Thysanoptera originating and diversifying in the late Permian (286 Ma, [110,111]). Finally our results are in agreement with paleobotanical evidence indicating that cycads first appeared in the late Permian and became more abundant and diverse in the Triassic [33,39,40].

Cycads proliferated during the Mesozoic, an era characterized by an increased occurrence and diversity of fossil cycads, broadly distributed throughout the relatively uniform climate of the supercontinent Pangaea [42]. Notably, the Triassic record of Cycadales is among the most well-known of cycad history, not only because of the marked increase in the number of definitive cycad taxa, but also because some of the most well-preserved and informative taxa are from this period [70]. It is generally accepted that cycads first became significant components of terrestrial floras during the Late Triassic as part of a global floral turnover with primitive seed-plant floras being replaced by conifers and cycads after the Permian-Triassic mass extinction (252.2 Ma) [40,112,113]. Cycads are generally believed to have reached a diversity peak during the Jurassic, and remained relatively stable in terms of diversity during the Cretaceous [34,112]. Our results further show that major lineages of extant cycads were established during the Early Cretaceous through to the Late Cretaceous, particularly in the Zamiaceae. The current diversity of Zamiaceae stems from surviving lineages of the Cretaceous, a period of rich fossil diversity for the clade [30,33,114-116].

### Global diversification pattern of cycads

The cycad timetree is remarkable for its long branches subtending the genus radiations. The most remarkable example is the branch leading to the genus *Cycas* that is more than 200 million years long. Our results indicate that extant diversification of cycad species occurred in the Neogene, in agreement with other studies [6,38,43]. No crown node of any modern species-rich genus of cycad has an age >19 Ma (credibility intervals extend back to 33 and 29 Ma for *Ceratozamia* and *Cycas*). Therefore, the ancient stem divergences of genera are decoupled from their recent crown group radiations. This decoupling can be explained by three hypotheses: *(i)* the long branches represent periods of low diversity, *(ii)* the long branches may be the result of periods of extinction [6,38,107,117], but it is unknown whether these were few and sudden or highly protracted, or *(iii)* the presence of ghost lineages, which are lineages that have temporal and/or geographic gaps in their fossil record (e.g. the Cenozoic fossil record of coelacanths). Our results are consistent with the second hypothesis because we show *(i)* quasi-synchronous and independent shifts in diversification at the crown (or near) of genus radiations that may be interpreted as the recovery period after an extinction event [107,117], and *(ii)* high rates of extinction along the long branches of the genus stems that may have extirpated entire lineages [32,33,59]. Our results, as well as fossil evidence [30-33,39,40,42,114-116], support the role of a mass extinction in shaping the cycad timetree, perhaps in the Late Cretaceous when BAMM extinction rates are elevated.

We found periods of extinction along the long branches of the tree. It is important to mention that these inferred periods of extinction are congruent with fossil data also indicating significant extinctions for the clade. From the Jurassic through the Paleogene, the rate of extinction was high for all lineages. The monotypic genera (*Microcycas* and *Stangeria*) and species-poor genera (*Bowenia*, *Dioon*, and *Lepidozamia*) were particularly impacted by extinction (Figure 6). Fossil data and cladistic analyses suggest that, for instance, the lineage today represented by *Stangeria* might have been more diverse in the past [32,59,118]. The surviving cycads re-diversified from the mid- to late Miocene when five lineages, isolated on different continents, underwent successful independent increases in diversification rate. Therefore our results favour the hypothesis of few and sudden extinction events. As potential explanations, this diversification pattern may be attributed to *(i)* competition with angiosperms (e.g. [34,35,112,113,119]), *(ii)* changes in, and final extinction of, non-avian dinosaur faunas that may have acted as seed dispersers (for Mesozoic extinction) [41], and/or *(iii)* global climate change that led to latitudinal shifts of the main vegetation belts and the elimination of cycads from higher latitudes of Eurasia, North America, and Patagonia [6,110,120], perhaps around the Eocene-Oligocene boundary that marked a significant shift from greenhouse to icehouse climate [38,121].

Discussions on cycad diversity through time are partly hampered due to the difficulty of distinguishing cycad foliage and pollen from that of the extinct (and phylogenetically distinct) clade Bennettitales [39,42]; most previous published diversity curves for Mesozoic plants do not separate Cycadales and Bennettitales, with two clades being grouped together as ‘cycadophytes’ (e.g. [34,39,112]). Also, the Cretaceous and Cenozoic cycad fossil record is probably too limited to enable understanding of the role of extinction, notably around the Cretaceous-Paleogene boundary. Although their apparent post mid-Cretaceous decline is recognized, we do not have quantitative evidence of the diversity decline nor do we know whether Cenozoic cycad diversity remained low until the Neogene radiations, or whether substantial early to mid-Cenozoic diversity existed but was affected by major extinctions. Besides illustrating the potential effect of BPP choice in Bayesian molecular dating, our study re-examines the dating of the cycads, proving a robust phylogenetic framework for future studies aimed at estimating how the diversity of cycads varied through geological time.

## Conclusions

Our study highlights that the branching process prior (or tree prior) in Bayesian molecular dating has an important effect on age estimates for cycads. The birth-death process had a better fit than the traditionally used Yule process as determined by marginal likelihood estimates and Bayes factors. It is likely that cycads are not an isolated case and we advocate for a closer investigation of the branching process prior, BPP, in all future molecular phylogenetic dating studies. At minimum we suggest conducting a similar model selection as we did for cycads to select the best-fitting prior and thus the best timetree. However, the birth-death process will not necessarily always better fit the data just because it seems more biologically realistic than a simpler Yule model, especially for shallower divergence times (i.e. recent clades). For instance, two recent studies on Australian diving beetles [106] and St. John’s wort [122] did not show any age differences using different BPPs, and none of the BPP was supported with Bayes factors; both clades originated in the Oligocene (*ca.* 34-28 Ma). On the contrary for deeper divergence times, age differences might be revealed when comparing a birth-death and a Yule process as exemplified in a dating study of darkling beetles that originated in the Jurassic (≈180 Ma) [27], but those differences were not as marked as in cycads. Based on these studies and our results, we expect that age differences deriving from different BPPs increase when clade age is very old (e.g. Triassic and backward).

As we gain greater understanding of the priors and parameters associated with molecular dating, there have been accordingly advances in our methodology. We note that other avenues for molecular dating are being developed because the standard dating procedure results in overlaying two prior distributions for a calibration node: one from the tree prior and one from the calibration prior [19,21,102]. In the last years, there are new methods that allow the inclusion of fossils in divergence time estimation as non-contemporaneous terminal tips rather than as node calibration points, also known as tip dating [123]. Recently, Heath *et al.* [103] introduced the fossilized birth–death process, a model for calibrating divergence time estimates in a Bayesian framework, explicitly acknowledging that extant species and fossils are part of the same macroevolutionary process. They argued that a single model that acts as a prior on the speciation times for both calibrated and uncalibrated nodes is a better representation of the lineage diversification process. The approach seems promising and will increasingly be used in future studies, like the application on the royal ferns (family Osmundaceae) [124]. The fossilized birth–death process best fitted the Osmundaceae fossil record and also provided speciation and extinction rates associated with the dated phylogeny.

## Availability of supporting data

The data set supporting the results of this article is available in the Dryad repository, http://dx.doi.org/10.5061/dryad.20k7m (see ref. [125]).

## Additional files

Additional file 1: Table S1. Taxa sampled and GenBank accession numbers of sequences used in the analyses. All PHYP sequences were generated by Nagalingum *et al.* [6], and sequences from matK and rbcL were downloaded from GenBank. “—” denotes sequence not available or voucher not indicated. Additional file 2: Figure S1. Phylogeny of Cycadales reconstructed (for 237 cycad species and three genes) using MrBayes and reversible jump MCMC. Posterior probabilities are shown at nodes. Only the nodes in the backbone are considered well-supported here. Nodes within generic radiations are generally below the standard threshold of robustness. The low node support within genera is likely due to low genetic divergence between species. Additional file 3: Figure S2. Convergence of the BAMM analysis with the chronogram reconstructed with a Yule prior. (A) The stationary of the MCMC before applying a burn-in. (B) The posterior distribution of number of shifts estimated before applying a burn-in. (C) The stationary of the MCMC after removing the burn-in phase. (D) The posterior distribution of number of shifts estimated after removing the burn-in phase. Additional file 4: Figure S3. Convergence of the BAMM analysis with the chronogram reconstructed with a birth-death prior. (A) The stationary of the MCMC before applying a burn-in. (B) The posterior distribution of number of shifts estimated before applying a burn-in. (C) The stationary of the MCMC after removing the burn-in phase. (D) The posterior distribution of number of shifts estimated after removing the burn-in phase. Additional file 5: Figure S4. Frequency distribution of distinct macroevolutionary rate regimes estimated using BAMM and the tree reconstructed with the Yule prior. (A) Prior distribution of the number of distinct processes. (B) Posterior distribution of the number of distinct processes (including the root process) on the cycad phylogeny reconstructed with a Yule model. A one-process model outperforms a two-process model. Additional file 6: Figure S5. Frequency distribution of distinct macroevolutionary rate regimes estimated using BAMM and the tree reconstructed with the birth-death prior. (A) Prior distribution of the number of distinct processes. (B) Posterior distribution of the number of distinct processes (including the root process) on the cycad phylogeny reconstructed with a birth-death model. A one-process model outperforms a two-process model. Additional file 7: Figure S6. Credible set of configuration shifts of cycads inferred with BAMM using the (A) tree with the Yule prior, and the (B) tree dated with the birth-death prior. Phylogenies show the distinct shift configurations with the highest posterior probability. For each shift configuration, the locations of rate shifts are shown as red (rate increases) and blue (rate decreases) circles, with circle size proportional to the marginal probability of the shift. Text labels (e.g. f =0.33) denote the posterior probability of each shift configuration. Additional file 8: Figure S7. The best shift configuration inferred with BAMM using the (A) tree with the Yule prior, and the (B) tree dated with the birth-death prior. Analysis with the Yule prior indicated a single rate shift (indicated by red circle) near the crowns of *Cycas* and *Encephalartos*, whereas the analysis with the birth-death prior indicated four major shifts at the richest genera: *Cycas*, *Zamia*, *Encephalartos*, and *Macrozamia*. Additional file 9: Figure S8. Macroevolutionary cohort matrix for speciation of cycads using the birth-death prior. Each cell in the matrix is coded by a color denoting the pairwise probability that two species share a common macroevolutionary rate regime. The cycad phylogeny reconstructed with a birth-death process is shown for reference on the left and upper margins of each cohort matrix. At least five major cohorts can be identified that are the four most species-rich genera: *Cycas*, *Zamia*, *Encephalartos*, and *Macrozamia*. Additional file 10: Figure S9. Macroevolutionary cohort matrix for speciation of cycads using the Yule prior. Same legend as Additional file 9: Figure S8.

## Abbreviations

BPP: Branching process prior
BF: Bayes factor
BRC: Bayesian relaxed-clock
ESS: Effective sample size
FC: Fossil calibration
HME: Harmonic mean estimate
HPD: Height posterior density
Ma: Million years ago
MCMC: Markov chain Monte Carlo
MLE: Marginal likelihood estimate
PP: Posterior probability
PSRF: Potential scale reduction factor
SS: Stepping-stone
UCLD: Uncorrelated lognormal distribution clock model
