# Supplementary material for: Origin and diversification of living cycads: a cautionary tale on the impact of the branching process prior in Bayesian molecular dating
Source: BMC Evol Biol. 2015 Apr 17;15:65. doi: 10.1186/s12862-015-0347-8 (PMC4449600; doi:10.1186/s12862-015-0347-8)
Supplement: Additional file 1: Table S1. — Taxa sampled and GenBank accession numbers of sequences used in the analyses. All PHYP sequences were generated by Nagalingum et al. [6], and sequences from matK and rbcL were downloaded from GenBank. “—” denotes sequence not available or voucher not indicated. [file 12862_2015_347_MOESM1_ESM.doc]

**Table S1** Taxa sampled and GenBank accession numbers of sequences used in the analyses. All *PHYP* sequences were generated by Nagalingum *et al.* (2011), and sequences from *matK* and *rbcL* were downloaded from GenBank. “—” denotes sequence not available or voucher not indicated.

| **Family** | **Genus, species, Author** | **Collection, voucher** | ***PHYP*** | ***rbcL*** | ***matK*** |
| --- | --- | --- | --- | --- | --- |
| Cycadaceae | *Cycas aculeata* Hill & Hiêp | NY100783 | JN655891 | — | — |
| Cycadaceae | *Cycas apoa* Hill | Little and Stevenson, 1107 (FTG, NY) | JN655892 | — | — |
| Cycadaceae | *Cycas armstrongii* Miq*.* | Little and Stevenson, 946 (FTG, NY) | JN655893 | — | — |
| Cycadaceae | *Cycas basaltica* Gardner | Stanberg, 810376 (NSW) | JN655894 | — | — |
| Cycadaceae | *Cycas beddomei* Dyer | Singh, s.n. 2003 (NY) | JN655895 | — | — |
| Cycadaceae | *Cycas bougainvilleana* Hill | Little and Stevenson, 950 (FTG, NY) | JN655896 | — | — |
| Cycadaceae | *Cycas brachycantha* Hill *et al.* | Nguyen, 310 (HN) | JN655897 | — | — |
| Cycadaceae | *Cycas cairnsiana* Muell*.* | Little and Stevenson, 951 (FTG, NY) | JN655898 | — | — |
| Cycadaceae | *Cycas calcicola* Maconochie | Little and Stevenson, 952 (FTG, NY) | JN655899 | — | — |
| Cycadaceae | *Cycas chamaoensis* Hill | Little and Stevenson, 954 (FTG, NY) | JN655900 | — | — |
| Cycadaceae | *Cycas chamberlainii* Brown & Kienholz | Little and Stevenson, 1109 (FTG, NY) | JN655901 | — | — |
| Cycadaceae | *Cycas chevalieri* Leandri | Nguyen, s.n. [CC242] (HN) | JN655902 | — | — |
| Cycadaceae | *Cycas circinalis* L. | Little and Stevenson, 1099 (FTG, NY) | JN655903 | L12674 | AF410164 |
| Cycadaceae | *Cycas clivicola* Hill | Little and Stevenson, 955 (FTG, NY) | JN655904 | — | GQ203830 |
| Cycadaceae | *Cycas collina* Hill *et al.* | Nguyen, s.n. [CC239] (HN) | JN655905 | — | — |
| Cycadaceae | *Cycas condaoensis* Hill & Yang | Nguyen, 181 (HN) | JN655906 | — | — |
| Cycadaceae | *Cycas couttsiana* Hill | Little and Stevenson, 958 (FTG, NY) | JN655907 | — | — |
| Cycadaceae | *Cycas debaoensis* Zhong & Chen | Holzman, s.n. [CC216] (NY) | JN655908 | — | — |
| Cycadaceae | *Cycas diannanensis* Guan & Tao | Little and Stevenson, 960 (FTG, NY) | JN655909 | — | — |
| Cycadaceae | *Cycas dolichophylla* Hill *et al.* | Nguyen, s.n. [CC241] (HN) | JN655910 | — | — |
| Cycadaceae | *Cycas edentata* de Laub*.* | Little and Stevenson, 961 (FTG, NY) | JN655911 | — | — |
| Cycadaceae | *Cycas elongata* (Leandri) Yue Wang | Little and Stevenson, 962 (FTG, NY) | JN655912 | — | — |
| Cycadaceae | *Cycas ferruginea* Wei | NY-HN, 099 (HN, NY) | JN655913 | — | — |
| Cycadaceae | *Cycas fugax* Hill *et al.* | Nguyen, 319 (HN) | JN655914 | — | — |
| Cycadaceae | *Cycas guizhouensis* Lan & Zou | Little and Stevenson, 963 (FTG, NY) | JN655915 | — | GQ203831 |
| Cycadaceae | *Cycas hainanensis* Chen | Little and Stevenson, 964 (FTG, NY) | JN655916 | — | GQ203832 |
| Cycadaceae | *Cycas hongheensis* Yang & Yang | Little and Stevenson, 967 (FTG, NY) | JN655917 | — | — |
| Cycadaceae | *Cycas lindstromii* Yang *et al.* | Little and Stevenson, 968 (FTG, NY) | JN655918 | — | — |
| Cycadaceae | *Cycas litoralis* Hill | Little and Stevenson, 969 (FTG, NY) | JN655919 | — | — |
| Cycadaceae | *Cycas maconochiei* Chirgwin & Hill | Little and Stevenson, 970 (FTG, NY) | JN655920 | — | — |
| Cycadaceae | *Cycas macrocarpa* Griff*.* | Little and Stevenson, 971 (FTG, NY) | JN655921 | — | — |
| Cycadaceae | *Cycas media* Br*.* subsp *media* Hill | Little and Stevenson, 973 (FTG, NY) | JN655922 | — | AF410162 |
| Cycadaceae | *Cycas media* Br*.* subsp*. ensata* Hill | Little and Stevenson, 972 (FTG, NY) | JN655923 | — | — |
| Cycadaceae | *Cycas micholitzii* Dyer | Little and Stevenson, 974 (FTG, NY) | JN655924 | — | AB116591 |
| Cycadaceae | *Cycas micronesica* Hill | Little and Stevenson, 975 (FTG, NY) | JN655925 | EU016864 | EU016806 |
| Cycadaceae | *Cycas miquelii* Warburg | Little and Stevenson, 976 (FTG, NY) | JN655926 | — | — |
| Cycadaceae | *Cycas multipinnata* Chen & Yang | Little and Stevenson, 977 (FTG, NY) | JN655927 | — | AF410155 |
| Cycadaceae | *Cycas nongnoochiae* Hill | Little and Stevenson, 978 (FTG, NY) | JN655928 | — | — |
| Cycadaceae | *Cycas ophiolitica* Hill | Little and Stevenson, 980 (FTG, NY) | JN655929 | — | — |
| Cycadaceae | *Cycas panzhihuaensis* Zhou & Yang | Little and Stevenson, 982 (FTG, NY) | JN655930 | — | GQ203834 |
| Cycadaceae | *Cycas pectinata A* Buch.-Ham*.* | Little and Stevenson, 984 (FTG, NY) | JN655931 | — | AB076238 |
| Cycadaceae | *Cycas pectinata B* Buch.-Ham*.* | NY-HN, 007 [CC233] (NY, HN) | JN655932 | — | AB076238 |
| Cycadaceae | *Cycas petraea* Lindstr. & Hill | Little and Stevenson, 985 (FTG, NY) | JN655933 | — | — |
| Cycadaceae | *Cycas platyphylla* Hill | Little and Stevenson, 986 (FTG, NY) | JN655934 | — | — |
| Cycadaceae | *Cycas revoluta* Thunb*.* | Little and Stevenson, 987 (FTG, NY) | JN655935 | AF462411 | AB116583 |
| Cycadaceae | *Cycas riuminiana* Porte ex Regel | Little and Stevenson, 988 (FTG, NY) | JN655936 | — | — |
| Cycadaceae | *Cycas rumphii* Miq*.* | Little and Stevenson, 989 (FTG, NY) | JN655937 | AF394338 | AF410161 |
| Cycadaceae | *Cycas schumanniana* Lauterb*.* | Little and Stevenson, 990 (FTG, NY) | JN655938 | — | — |
| Cycadaceae | *Cycas seemannii* Braun | Little and Stevenson, 991 (FTG, NY) | JN655939 | AF394340 | — |
| Cycadaceae | *Cycas segmentifida* Yue Wang & Deng | Little and Stevenson, 992 (FTG, NY) | JN655940 | — | — |
| Cycadaceae | *Cycas semota* Hill | Little and Stevenson, 993 (FTG, NY) | JN655941 | — | — |
| Cycadaceae | *Cycas sexseminifera* Wei | Little and Stevenson, 994 (FTG, NY) | JN655942 | — | — |
| Cycadaceae | *Cycas siamensis* Miq*.* | Little and Stevenson, 995 (FTG, NY) | JN655943 | — | AF410165 |
| Cycadaceae | *Cycas simplicipinna* (Smitinand) Hill | Little and Stevenson, 996 (FTG, NY) | JN655944 | — | AB116587 |
| Cycadaceae | *Cycas sphaerica* Roxb*.* | Little and Stevenson, 1114 (FTG, NY) | JN655945 | — | — |
| Cycadaceae | *Cycas szechuanensis* Cheng *et al.* | Little and Stevenson, 997 (FTG, NY) | JN655946 | — | — |
| Cycadaceae | *Cycas taitungensis* Shen *et al.* | Little and Stevenson, 998 (FTG, NY) | JN655947 | — | AF279795 |
| Cycadaceae | *Cycas tanqingii* Yue Wang | Little and Stevenson, 999 (FTG, NY) | JN655948 | — | AB116584 |
| Cycadaceae | *Cycas tansachana* Hill & Yang | Little and Stevenson, 1000 (FTG, NY) | JN655949 | — | — |
| Cycadaceae | *Cycas thouarsii* Br. ex Gaudich*.* | Little and Stevenson, 1001 (FTG, NY) | JN655950 | AF394336 | AB116589 |
| Cycadaceae | *Cycas tropophylla* Hill & Loc | Nguyen, 001 (HN) | JN655951 | — | — |
| Cycadaceae | *Cycas wadei* Merrill | — | — | AF394341 | GQ203837 |
| Cycadaceae | *Cycas xipholepis* Hill | Little and Stevenson, 1116 (FTG, NY) | JN655952 | — | — |
| Cycadaceae | *Cycas yorkiana* Hill | Little and Stevenson, 1003 (FTG, NY) | JN655953 | — | — |
| Cycadaceae | *Cycas zeylanica* (Schust.) Lindstr. & Hill | Singh, s.n. 1990 (NY) | JN655954 | — | — |
| Zamiaceae | *Bowenia serrulata* (Bull) Chamb*.* | Little and Stevenson, 1004 (FTG, NY) | JN655955 | L12671 | AF410173 |
| Zamiaceae | *Bowenia spectabilis* | Little and Stevenson, 1005 (FTG, NY) | — | AF531202 | GQ203826 |
| Zamiaceae | *Ceratozamia alvarezii* Pérez-Farrera *et al.* | — | — | — | GU807321 |
| Zamiaceae | *Ceratozamia becerrae* Pérez-Farrera | — | — | — | GU807322 |
| Zamiaceae | *Ceratozamia chimalapensis* Pérez-Farr. & Vov. | — | — | — | GU807323 |
| Zamiaceae | *Ceratozamia decumbens* Vovides *et al.* | Stevenson, DWS4-2007/XAL2002-028 (NY, XAL) | JN655956 | — | GU807324 |
| Zamiaceae | *Ceratozamia euryphyllidia* Vázq.Torres *et al.* | — | — | — | GU807325 |
| Zamiaceae | *Ceratozamia fuscoviridis* Moore | Stevenson, DWS3-2007/XAL1999-055.1 (NY, XAL) | JN655957 | — | — |
| Zamiaceae | *Ceratozamia hildae* Landry & Wilson | — | — | JQ770262 | GQ203827 |
| Zamiaceae | *Ceratozamia huastecorum* Avendaño *et al.* | Stevenson, 1990-002A (NY) | JN655958 | — | GU807327 |
| Zamiaceae | *Ceratozamia kuesteriana* Regel | — | — | AF394347 | GQ203828 |
| Zamiaceae | *Ceratozamia latifolia* Miq*.* | Little and Stevenson, 1008 (FTG, NY) | JN655959 | — | GU807329 |
| Zamiaceae | *Ceratozamia matudae* Lundell | — | — | — | GU807330 |
| Zamiaceae | *Ceratozamia mexicana* Brongn. | Little and Stevenson, 1009 (FTG, NY) | JN655960 | AF394345 | AF279794 |
| Zamiaceae | *Ceratozamia microstrobila* Vovides & Rees | Little and Stevenson, 1010 (FTG, NY) | JN655961 | AF531210 | GQ203829 |
| Zamiaceae | *Ceratozamia miqueliana* Wendl*.* | Little and Stevenson, 1011 (FTG, NY) | JN655962 | AF462410 | GU807333 |
| Zamiaceae | *Ceratozamia mirandae* Vovides *et al.* | Little and Stevenson, 1012 (FTG, NY) | JN655963 | — | GU807334 |
| Zamiaceae | *Ceratozamia mixeorum* Chemnick *et al.* | Stevenson, DWS6-2007/XAL2000-027A (NY, XAL) | JN655964 | — | GU807335 |
| Zamiaceae | *Ceratozamia morettii* Vázq. *et al.* | Little and Stevenson, 1013 (FTG, NY) | JN655965 | — | GU807336 |
| Zamiaceae | *Ceratozamia norstogii* Stev. | — | — | AF531213 | GU807337 |
| Zamiaceae | *Ceratozamia robusta* Miq. | — | — | AF394346 | GU807338 |
| Zamiaceae | *Ceratozamia sabatoi* Vovides *et al.* | Little and Stevenson, 1015 (FTG, NY) | JN655966 | — | GU807339 |
| Zamiaceae | *Ceratozamia vovidesii* Pérez-Farr. & Iglesias | — | — | — | GU807340 |
| Zamiaceae | *Ceratozamia whitelockiana* Chemnick & Greg*.* | Stevenson, DWS1-200/XAL2001-034B (NY, XAL) | JN655967 | — | GU807341 |
| Zamiaceae | *Ceratozamia zaragozae* Medellin-Leal | Little and Stevenson, 1106 (FTG, NY) | JN655968 | — | GU807342 |
| Zamiaceae | *Ceratozamia zoquorum* Stev. | — | — | — | GU807343 |
| Zamiaceae | *Dioon califanoi* De Luca & Sabato | — | — | AF394353 | — |
| Zamiaceae | *Dioon edule* Lindl. | Little and Stevenson, 1092 (FTG, NY) | JN655970 | AF531203 | AB076193 |
| Zamiaceae | *Dioon mejiae* Standl. & Williams | Little and Stevenson, 1121 (FTG, NY) | — | — | GQ203839 |
| Zamiaceae | *Dioon merolae* De Luca *et al.* | Little and Stevenson, 1122 (FTG, NY) | JN655971 | — | — |
| Zamiaceae | *Dioon purpusii* Rose | Little and Stevenson, 1093 (FTG, NY) | JN655972 | AF394352 | — |
| Zamiaceae | *Dioon rzedowskii* De Luca *et al.* | Little and Stevenson, 1123 (FTG, NY) | — | — | GQ203841 |
| Zamiaceae | *Dioon spinulosum* Dyer & Eichler | Little and Stevenson, 1094 (FTG, NY) | JN655973 | AF394351 | AB076228 |
| Zamiaceae | *Dioon tomasellii* De Luca *et al.* | Little and Stevenson, 1125 (FTG, NY) | JN655974 | AF531204 | AB076230 |
| Zamiaceae | *Encephalartos aemulans* Vorster | Little and Stevenson, 1018 (FTG, NY) | JN655975 | — | — |
| Zamiaceae | *Encephalartos altensteinii* Lehm. | Little and Stevenson, 1019 (FTG, NY) | JN655976 | — | — |
| Zamiaceae | *Encephalartos aplanatus* Vorster | Little and Stevenson, 1126 (FTG, NY) | JN655977 | AY335241 | — |
| Zamiaceae | *Encephalartos arenarius* Dyer | Little and Stevenson, 1020 (FTG, NY) | JN655978 | AF531206 | — |
| Zamiaceae | *Encephalartos barteri* Carruth. ex Miq*.* | Little and Stevenson, 1095 (FTG, NY) | JN655979 | AF462413 | AB076203 |
| Zamiaceae | *Encephalartos barteri allochrous* Newton | PR892 | — | AF394358 | JQ046255 |
| Zamiaceae | *Encephalartos barteri barteri* Carruth. ex Miq. | PR878 | — | JQ025457 | JQ046256 |
| Zamiaceae | *Encephalartos brevifoliolatus* Vorster | Xdk2 | — | JQ025459 | JQ046253 |
| Zamiaceae | *Encephalartos bubalinus* Melville | Little and Stevenson, 1021 (FTG, NY) | JN655980 | — | — |
| Zamiaceae | *Encephalartos caffer* (Thunb.) Lehm. | Little and Stevenson, 1101 (FTG, NY) | JN655981 | — | — |
| Zamiaceae | *Encephalartos cerinus* Lavranos & Goode | Little and Stevenson, 1022 (FTG, NY) | JN655982 | AY335256 | — |
| Zamiaceae | *Encephalartos chimanimaniensis* Dyer &.Verd. | PR888 | — | JQ025476 | JQ046247 |
| Zamiaceae | *Encephalartos concinnus* Dyer & Verd*.* | Little and Stevenson, 1023 (FTG, NY) | JN655983 | — | — |
| Zamiaceae | *Encephalartos cupidus* Dyer | Little and Stevenson, 1127 (FTG, NY) | JN655984 | AY335250 | — |
| Zamiaceae | *Encephalartos cycadifolius* (Jacq.) Lehm. | Little and Stevenson, 1128 (FTG, NY) | JN655985 | AF394369 | — |
| Zamiaceae | *Encephalartos dolomiticus* Lavranos & Goode | Sass, 119 (UC) | JN655986 | — | — |
| Zamiaceae | *Encephalartos dyerianus* Lavranos & Goode | Sass, 128 (UC) | JN655987 | AY335251 | — |
| Zamiaceae | *Encephalartos equatorialis* Hurter | PR900 | — | JQ025494 | JQ046239 |
| Zamiaceae | *Encephalartos eugene-maraisii* Verd. | Little and Stevenson, 1024 (FTG, NY) | JN655988 | AF394357 | — |
| Zamiaceae | *Encephalartos ferox* Bertol. f. | Little and Stevenson, 1025 (FTG, NY) | JN655989 | AY335243 | — |
| Zamiaceae | *Encephalartos friderici-guilielmi* Lehm. | Sass, 129 (UC) | JN655990 | — | — |
| Zamiaceae | *Encephalartos ghellinckii* Lem*.* | Sass, 124 (UC) | JN655991 | AY335247 | — |
| Zamiaceae | *Encephalartos gratus* Prain | Little and Stevenson, 1026 (FTG, NY) | JN655992 | AF394361 | AB076207 |
| Zamiaceae | *Encephalartos heenanii* Dyer | PR776 | — | JQ025524 | JQ046228 |
| Zamiaceae | *Encephalartos hildebrandtii* Braun & Bouché | Little and Stevenson, 1027 (FTG, NY) | JN655993 | AF394360 | — |
| Zamiaceae | *Encephalartos hirsutus* Hurter | PR718 | — | JQ025534 | JQ046226 |
| Zamiaceae | *Encephalartos horridus* (Jacq.) Lehm. | Little and Stevenson, 1028 (FTG, NY) | JN655994 | AF394366 | AF410169 |
| Zamiaceae | *Encephalartos humilis* Verd*.* | Sass, 116 (UC) | JN655995 | AF394363 | — |
| Zamiaceae | *Encephalartos inopinus* Dyer | Little and Stevenson, 1130 (FTG, NY) | JN655996 | AY335254 | — |
| Zamiaceae | *Encephalartos ituriensis* Bamps & Lisowski | Little and Stevenson, 1131 (FTG, NY) | JN655997 | — | — |
| Zamiaceae | *Encephalartos kanga* Pócs & Luke | PR907 | — | — | JQ046298 |
| Zamiaceae | *Encephalartos kisambo* Faden & Beentje | Little and Stevenson, 1132 (FTG, NY) | JN655998 | AY335252 | — |
| Zamiaceae | *Encephalartos laevifolius* Stapf & Burtt Davy | Sass, s.n. [UC2005.0862] (UC) | JN655999 | AF394370 | — |
| Zamiaceae | *Encephalartos lanatus* Stapf & Burtt Davy | Little and Stevenson, 1133 (FTG, NY) | JN656000 | AY335248 | — |
| Zamiaceae | *Encephalartos latifrons* Lehm. | PR806 | — | JQ025565 | JQ046296 |
| Zamiaceae | *Encephalartos laurentianus* De Wild*.* | Little and Stevenson, 1029 (FTG, NY) | JN656001 | — | — |
| Zamiaceae | *Encephalartos lebomboensis* Verd*.* | Little and Stevenson, 1030 (FTG, NY) | JN656002 | — | — |
| Zamiaceae | *Encephalartos lehmannii* Lehm. | Little and Stevenson, 1031 (FTG, NY) | JN656003 | AY335259 | — |
| Zamiaceae | *Encephalartos longifolius* (Jacq.) Lehm. | Little and Stevenson, 1032 (FTG, NY) | JN656004 | AF394365 | AB076210 |
| Zamiaceae | *Encephalartos macrostrobilus* Jones & Wynants | Sass, 122 (UC) | JN656005 | — | — |
| Zamiaceae | *Encephalartos manikensis* (Gilliland) Gilliland | Little and Stevenson, 1033 (FTG, NY) | JN656006 | AY335255 | — |
| Zamiaceae | *Encephalartos marunguensis* Devred | PR912 | — | — | JQ046200 |
| Zamiaceae | *Encephalartos middelburgensis* Vorster | Sass, 115 (UC) | JN656007 | — | — |
| Zamiaceae | *Encephalartos msinganus* Vorster | Little and Stevenson, 1034 (FTG, NY) | JN656008 | — | — |
| Zamiaceae | *Encephalartos munchii* Dyer & Verd*.* | Little and Stevenson, 1134 (FTG, NY) | JN656009 | AF394356 | — |
| Zamiaceae | *Encephalartos natalensis* Dyer & Verd*.* | Little and Stevenson, 1035 (FTG, NY) | JN656010 | AY335242 | — |
| Zamiaceae | *Encephalartos ngoyanus* Verd*.* | Little and Stevenson, 1135 (FTG, NY) | JN656011 | AY335240 | — |
| Zamiaceae | *Encephalartos nubimontanus* Hurter | Sass, 121 (UC) | JN656012 | — | — |
| Zamiaceae | *Encephalartos paucidentatus* Stapf & Burtt Davy | Little and Stevenson, 1036 (FTG, NY) | JN656013 | — | — |
| Zamiaceae | *Encephalartos poggei* Asch. | PR911 | — | — | JQ046282 |
| Zamiaceae | *Encephalartos princeps* Dyer | Little and Stevenson, 1037 (FTG, NY) | JN656014 | AY335246 | — |
| Zamiaceae | *Encephalartos pterogonus* Dyer & Verd*.* | Little and Stevenson, 1136 (FTG, NY) | JN656015 | AF394368 | — |
| Zamiaceae | *Encephalartos schaijesii* Malaisse *et al.* | Sass, 125 (UC) | JN656016 | — | — |
| Zamiaceae | *Encephalartos schmitzii* Malaisse | Little and Stevenson, 1137 (FTG, NY) | JN656017 | — | — |
| Zamiaceae | *Encephalartos sclavoi* De Luca *et al.* | Little and Stevenson, 1038 (FTG, NY) | JN656018 | — | — |
| Zamiaceae | *Encephalartos senticosus* Vorster | Little and Stevenson, 1039 (FTG, NY) | JN656019 | AY335244 | — |
| Zamiaceae | *Encephalartos septentrionalis* Schweinf*.* | Little and Stevenson, 1138 (FTG, NY) | JN656020 | AF394359 | — |
| Zamiaceae | *Encephalartos tegulaneus* Melville | Little and Stevenson, 1040 (FTG, NY) | JN656021 | — | — |
| Zamiaceae | *Encephalartos transvenosus* Stapf & Burtt Davy | Little and Stevenson, 1041 (FTG, NY) | JN656022 | GQ248599 | — |
| Zamiaceae | *Encephalartos trispinosus* (Hook.) Dyer | Little and Stevenson, 1043 (FTG, NY) | JN656023 | AF394367 | — |
| Zamiaceae | *Encephalartos turneri* Lavranos & Goode | Little and Stevenson, 1044 (FTG, NY) | JN656024 | — | — |
| Zamiaceae | *Encephalartos umbeluziensis* Dyer | Little and Stevenson, 1046 (FTG, NY) | — | AY335257 | GQ203848 |
| Zamiaceae | *Encephalartos villosus* Lem*.* | Little and Stevenson, 1047 (FTG, NY) | JN656025 | AY335261 | — |
| Zamiaceae | *Encephalartos whitelockii* Hurter | Little and Stevenson, 1048 (FTG, NY) | JN656026 | AY335258 | — |
| Zamiaceae | *Encephalartos woodii* Sander | Little and Stevenson, 1139 (FTG, NY) | JN656027 | AY335245 | — |
| Zamiaceae | *Lepidozamia hopei* Regel | Little and Stevenson, 1049 (FTG, NY) | JN656028 | AF394342 | AB076212 |
| Zamiaceae | *Lepidozamia peroffskyana* Regel | Little and Stevenson, 1050 (FTG, NY) | JN656029 | AF531208 | AB076213 |
| Zamiaceae | *Macrozamia communis* Johnson | — | AJ286643 | AF531205 | AB076215 |
| Zamiaceae | *Macrozamia crassifolia* Forst. & Jones | Little and Stevenson, 1166 (FTG, NY) | JN656030 | — | — |
| Zamiaceae | *Macrozamia diplomera* (Muell.) Johnson | Pell, 1987-315 (LSU) | JN656031 | — | — |
| Zamiaceae | *Macrozamia douglasii* Hill ex Bailey | Little and Stevenson, 1141 (FTG, NY) | JN656032 | — | — |
| Zamiaceae | *Macrozamia dyeri* (Muell.) Gardner | Little and Stevenson, 1052 (FTG, NY) | JN656033 | — | AF410168 |
| Zamiaceae | *Macrozamia elegans* Hill & Jones | Stanberg, 79 (NSW) | JN656034 | — | — |
| Zamiaceae | *Macrozamia flexuosa* Moore | Pell, GWL0328 (LSU) | JN656035 | — | — |
| Zamiaceae | *Macrozamia fraseri* Miq*.* | Little and Stevenson, 1053 (FTG, NY) | JN656036 | — | — |
| Zamiaceae | *Macrozamia glaucophylla* Jones | Sass, 123 (UC) | JN656037 | — | — |
| Zamiaceae | *Macrozamia johnsonii* Jones & Hill | Little and Stevenson, 1054 (FTG, NY) | JN656038 | — | — |
| Zamiaceae | *Macrozamia lomandroides* Jones | Pell, 1993-174 (LSU) | JN656039 | — | — |
| Zamiaceae | *Macrozamia lucida* Johnson | Little and Stevenson, 1056 (FTG, NY) | JN656040 | AF394344 | AB076218 |
| Zamiaceae | *Macrozamia macdonnellii* (Muell. ex Miq.) DC*.* | Little and Stevenson, 1057 (FTG, NY) | JN656041 | — | — |
| Zamiaceae | *Macrozamia miquelii* (Muell.) DC. | Little and Stevenson, 1058 (FTG, NY) | JN656042 | — | — |
| Zamiaceae | *Macrozamia montana* Hill | Little and Stevenson, 1143 (FTG, NY) | JN656043 | — | — |
| Zamiaceae | *Macrozamia moorei* Muell*.* | Little and Stevenson, 1059 (FTG, NY) | JN656044 | AF394343 | AB076220 |
| Zamiaceae | *Macrozamia mountperriensis* Bailey | Little and Stevenson, 1144 (FTG, NY) | JN656045 | — | — |
| Zamiaceae | *Macrozamia pauli-guilielmi* Hill & Muell*.* | Little and Stevenson, 1096 (FTG, NY) | JN656046 | — | — |
| Zamiaceae | *Macrozamia platyrhachis* Bailey | Pell, 1996-296 (LSU) | JN656047 | — | — |
| Zamiaceae | *Macrozamia plurinervia* (Johnson) Jones | Little and Stevenson, 1060 (FTG, NY) | JN656048 | — | — |
| Zamiaceae | *Macrozamia polymorpha* Jones | Little and Stevenson, 1146 (FTG, NY) | JN656049 | — | — |
| Zamiaceae | *Macrozamia reducta* Hill & Jones | Stanberg, 75 (NSW) | JN656050 | — | — |
| Zamiaceae | *Macrozamia riedlei* (Gaudich.) Gardner | Stevenson, 1593/87A (NY) | JN656051 | — | — |
| Zamiaceae | *Macrozamia serpentine* Jones & Forst*.* | Little and Stevenson, 1062 (FTG, NY) | JN656052 | — | — |
| Zamiaceae | *Macrozamia spiralis* (Salisb.) Miq*.* | Stevenson, 1594/87A (NY) | JN656053 | — | — |
| Zamiaceae | *Macrozamia stenomera* Johnson | Sass, 117 (UC) | JN656054 | — | — |
| Zamiaceae | *Microcycas calocoma* (Miq.) DC*.* | Little and Stevenson, 1063 (FTG, NY) | JN656055 | AF531214 | AB076194 |
| Zamiaceae | *Stangeria eriopus* (Kunze) Baill*.* | Little and Stevenson, 1006 (FTG, NY) | JN656056 | DQ646007 | AB076201 |
| Zamiaceae | *Zamia acuminata* Oers. ex Dyer | Little and Stevenson, 1064 (FTG, NY) | JN656057 | — | — |
| Zamiaceae | *Zamia amblyphyllidia* Stev*.* | Little and Stevenson, 1065 (FTG, NY) | JN656058 | — | — |
| Zamiaceae | *Zamia angustifolia* Jacq*.* | Little and Stevenson, 1066 (FTG, NY) | JN656059 | — | AB076567 |
| Zamiaceae | *Zamia chigua* Seem*.* | Little and Stevenson, 1068 (FTG, NY) | JN656060 | — | — |
| Zamiaceae | *Zamia cremnophila* Vovides *et al.* | — | — | — | GU807344 |
| Zamiaceae | *Zamia dressleri* Stev*.* | Little and Stevenson, 1148 (FTG, NY) | JN656061 | — | — |
| Zamiaceae | *Zamia elegantissima* Schutzman *et al.* | Little and Stevenson, 1168 (FTG, NY) | JN656062 | — | — |
| Zamiaceae | *Zamia fairchildiana* Gómez | Stevenson, MBC20070832 (NY) | JN656063 | — | — |
| Zamiaceae | *Zamia fischeri* Miq. | — | — | AF531216 | — |
| Zamiaceae | *Zamia furfuracea A* L. f*.* | Little and Stevenson, 1098 (FTG, NY) | JN656064 | AF202959 | AF410170 |
| Zamiaceae | *Zamia furfuracea B* L. f*.* | Pell, s.n. [CC288] (LSU) | JN656065 | — | — |
| Zamiaceae | *Zamia herrerae* Calderón & Standl. | — | — | — | GU807346 |
| Zamiaceae | *Zamia hymenophyllidia* Stev*.* | Little and Stevenson, 1151 (FTG, NY) | JN656066 | — | — |
| Zamiaceae | *Zamia inermis* Vovides *et al.* | — | — | L12683 | GQ203860 |
| Zamiaceae | *Zamia integrifolia* L*.* f. | Turnbull, FA62291A (NY) | JN656067 | — | — |
| Zamiaceae | *Zamia ipetiensis* Stev*.* | Little and Stevenson, 1071 (FTG, NY) | JN656068 | — | — |
| Zamiaceae | *Zamia katzeriana* Regel | Little and Stevenson, 1164 (FTG, NY) | JN656069 | — | — |
| Zamiaceae | *Zamia kickxii* Miq. | Little and Stevenson, 1072 (FTG, NY) | JN656070 | — | — |
| Zamiaceae | *Zamia lacandona* Schutzman & Vovides | Little and Stevenson, 1073 (FTG, NY) | JN656071 | — | — |
| Zamiaceae | *Zamia lawsoniana* Dyer | Stevenson, DWS5-2007/XAL2000-049A (NY, XAL) | JN656072 | — | — |
| Zamiaceae | *Zamia lecointei* Ducke | Little and Stevenson, 1152 (FTG, NY) | JN656073 | — | — |
| Zamiaceae | *Zamia lindenii* Regel & André | — | — | AF531219 | — |
| Zamiaceae | *Zamia loddigesii* Miq*.* | Little and Stevenson, 1074 (FTG, NY) | JN656074 | — | — |
| Zamiaceae | *Zamia manicata* Linden ex Regel | Little and Stevenson, 1153 (FTG, NY) | JN656075 | — | — |
| Zamiaceae | *Zamia muricata* Willd*.* | Little and Stevenson, 1075 (FTG, NY) | JN656076 | — | AB076187 |
| Zamiaceae | *Zamia neurophyllidia* Stev*.* | Stevenson, 1255 (NY) | JN656077 | — | — |
| Zamiaceae | *Zamia obliqua* Braun | Little and Stevenson, 1154 (FTG, NY) | JN656078 | — | — |
| Zamiaceae | *Zamia paucijuga* Wieland | Little and Stevenson, 1077 (FTG, NY) | JN656079 | AF531220 | — |
| Zamiaceae | *Zamia picta* Dyer | Little and Stevenson, 1156 (FTG, NY) | JN656080 | — | — |
| Zamiaceae | *Zamia poeppigiana* Mart. & Eichler | Little and Stevenson, 1157 (FTG, NY) | JN656081 | AF531219 | — |
| Zamiaceae | *Zamia polymorpha* Stev. | — | — | — | GU807349 |
| Zamiaceae | *Zamia portoricensis* Urban | Little and Stevenson, 1079 (FTG, NY) | JN656082 | — | AB076189 |
| Zamiaceae | *Zamia pseudomonticola* Gómez | Little and Stevenson, 1081 (FTG, NY) | JN656083 | — | — |
| Zamiaceae | *Zamia pumila* L. | Little and Stevenson, 1100 (FTG, NY) | JN656084 | AY056557 | — |
| Zamiaceae | *Zamia purpurea* Vovides *et al.* | Little and Stevenson, 1160 (FTG, NY) | JN656085 | — | — |
| Zamiaceae | *Zamia pygmaea* Sims | Turnbull, s.n. [CC253] (NY) | JN656086 | — | — |
| Zamiaceae | *Zamia restrepoi* Stev. | Sass, s.n. [UC2006.0036] (UC) | JN655969 | — | — |
| Zamiaceae | *Zamia skinneri* Warsz. ex Dietrich | Little and Stevenson, 1083 (FTG, NY) | JN656087 | AF531221 | — |
| Zamiaceae | *Zamia spartea* DC*.* | Little and Stevenson, 1087 (FTG, NY) | JN656088 | AF394350 | — |
| Zamiaceae | *Zamia splendens* Schutzman | — | — | AF394348 | — |
| Zamiaceae | *Zamia standleyi* Schutzman | Little and Stevenson, 1162 (FTG, NY) | JN656089 | — | — |
| Zamiaceae | *Zamia variegata* Warsz*.* | Little and Stevenson, 1155 (FTG, NY) | JN656090 | — | — |
| Zamiaceae | *Zamia vazquezii* Stev. *et al.* | Little and Stevenson, 1088 (FTG, NY) | — | — | GQ203868 |
| Ginkgoaceae | *Ginkgo biloba* L. | Mathews 461 (A) | JN656091 | DQ069500 | DQ069584 |
| Araucariaceae | *Araucaria heterophylla* (Salisb.) Franco | Mathews 609 (A) | JN656092 | U96462 | AF456374 |
| Cupressaceae | *Cryptomeria japonica* (Thunb. ex L.f.) Don | Arnold Arboretum 838-53-A | JN656093 | AJ621937 | AB023984 |
| Pinaceae | *Abies firma* Siebl. & Zucc*.* | Arnold Arboretum 1043-74-C | JN656094 | AB015647 | AF143436 |
| Pinaceae | *Pinus strobus* L. | Mathews 477 (A) | JN656095 | AY497219 | AY497255 |
| Pinaceae | *Pseudotsuga menziesii* (Mirb.) Franco | Mathews 510 (A) | JN656096 | AY664856 | AF143439 |
