# Supplementary figures and images for: Origin and diversification of living cycads: a cautionary tale on the impact of the branching process prior in Bayesian molecular dating
Source: BMC Evol Biol. 2015 Apr 17;15:65. doi: 10.1186/s12862-015-0347-8 (PMC4449600; doi:10.1186/s12862-015-0347-8)

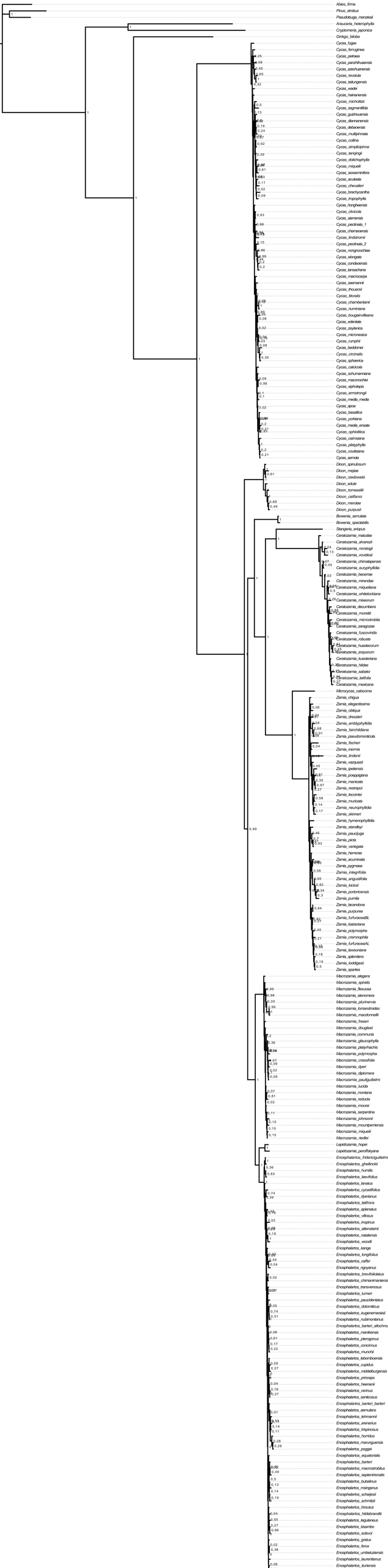

Supplement: Additional file 2: Figure S1. — Phylogeny of Cycadales reconstructed (for 237 cycad species and three genes) using MrBayes and reversible jump MCMC. Posterior probabilities are shown at nodes. Only the nodes in the backbone are considered well-supported here. Nodes within generic radiations are generally below the standard threshold of robustness. The low node support within genera is likely due to low genetic divergence between species. [file 12862_2015_347_MOESM2_ESM.pdf]

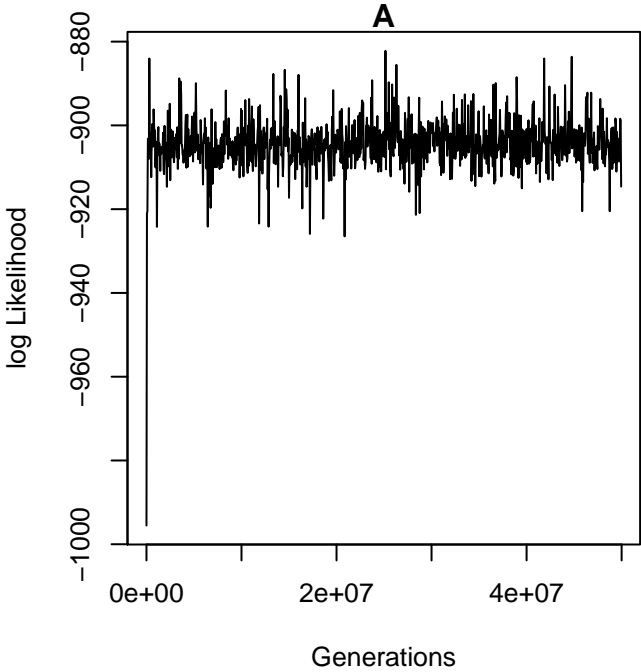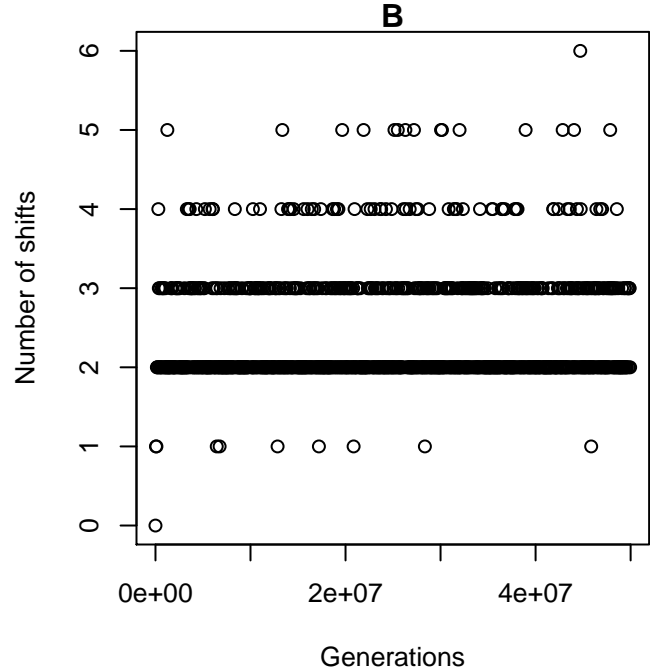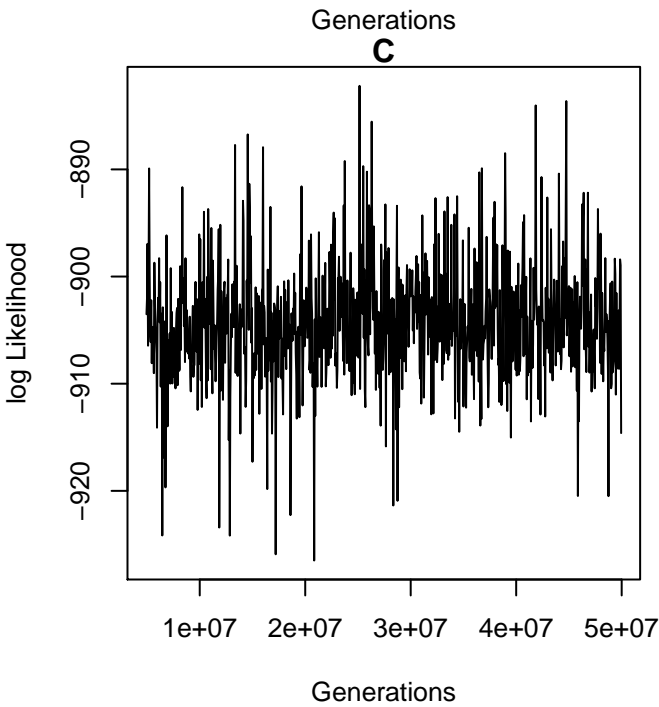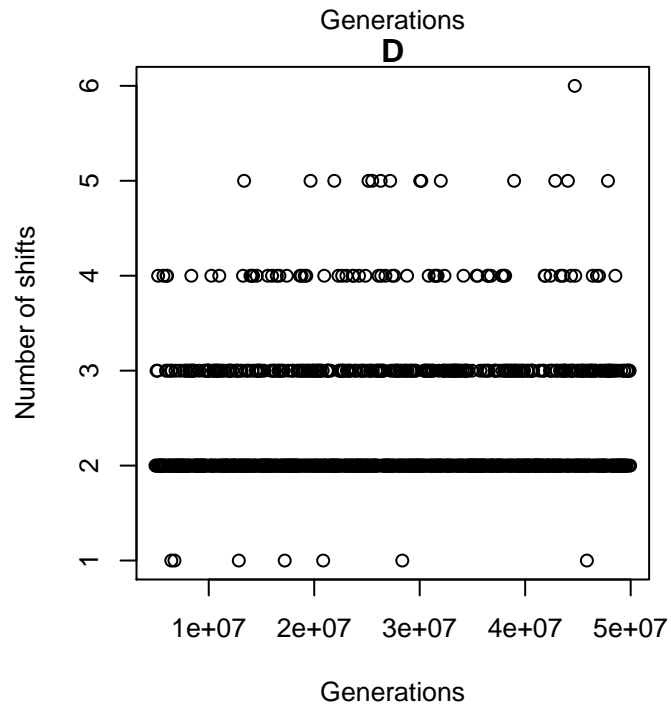

Supplement: Additional file 3: Figure S2. — Convergence of the BAMM analysis with the chronogram reconstructed with a Yule prior. (A) The stationary of the MCMC before applying a burn-in. (B) The posterior distribution of number of shifts estimated before applying a burn-in. (C) The stationary of the MCMC after removing the burn-in phase. (D) The posterior distribution of number of shifts estimated after removing the burn-in phase. [file 12862_2015_347_MOESM3_ESM.pdf]

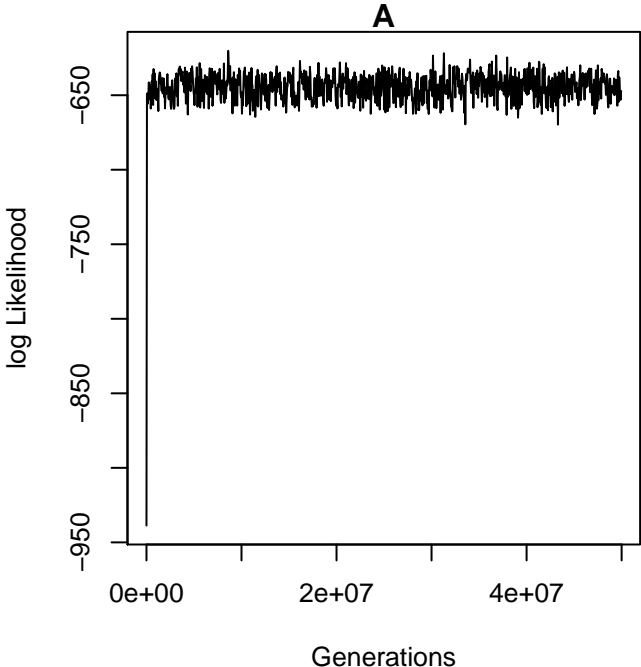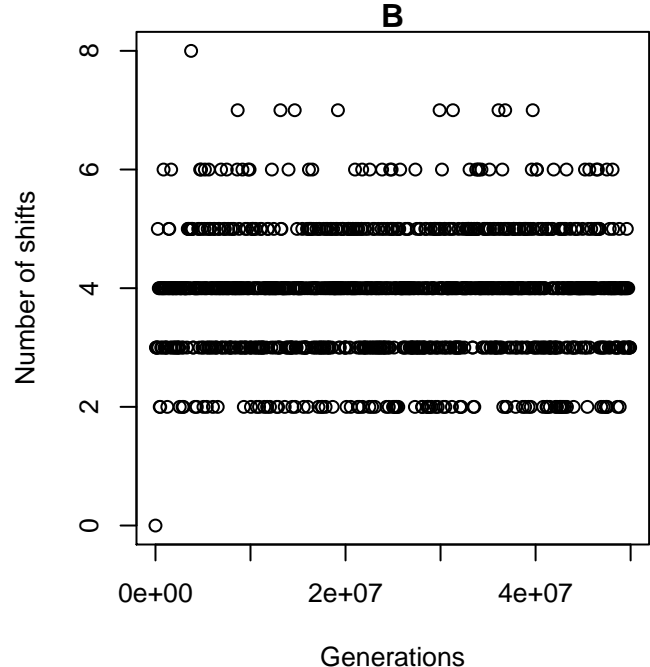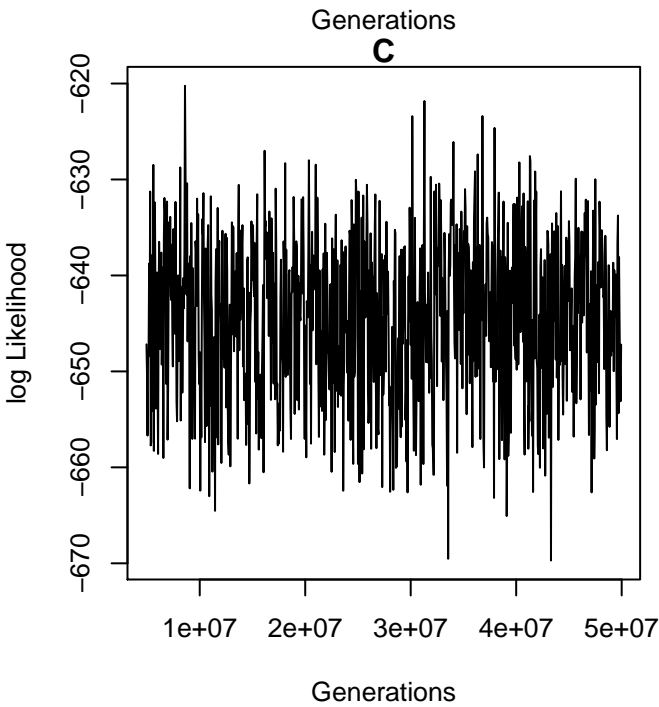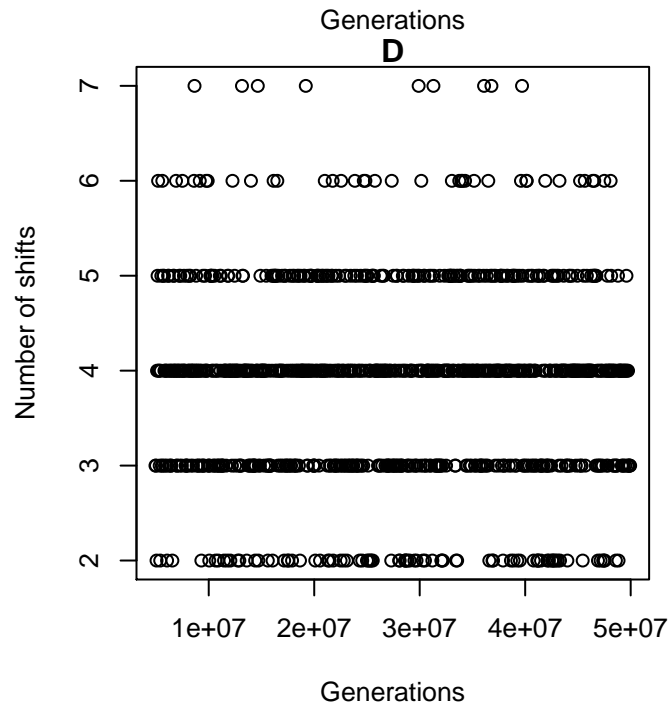

Supplement: Additional file 4: Figure S3. — Convergence of the BAMM analysis with the chronogram reconstructed with a birth-death prior. (A) The stationary of the MCMC before applying a burn-in. (B) The posterior distribution of number of shifts estimated before applying a burn-in. (C) The stationary of the MCMC after removing the burn-in phase. (D) The posterior distribution of number of shifts estimated after removing the burn-in phase. [file 12862_2015_347_MOESM4_ESM.pdf]

**A**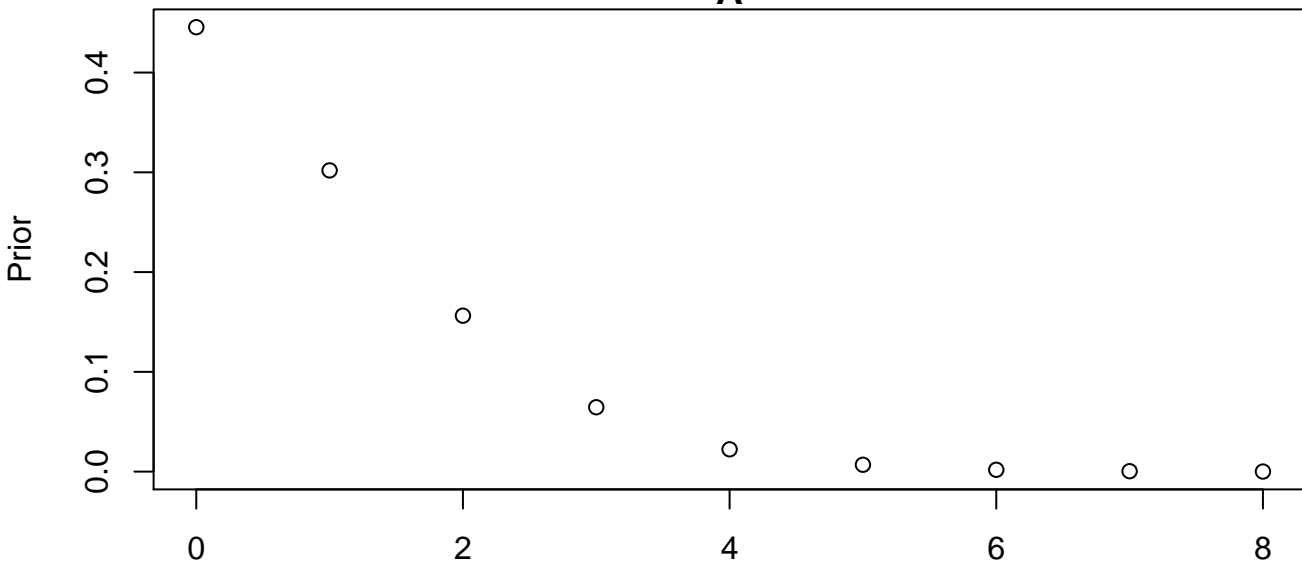**B**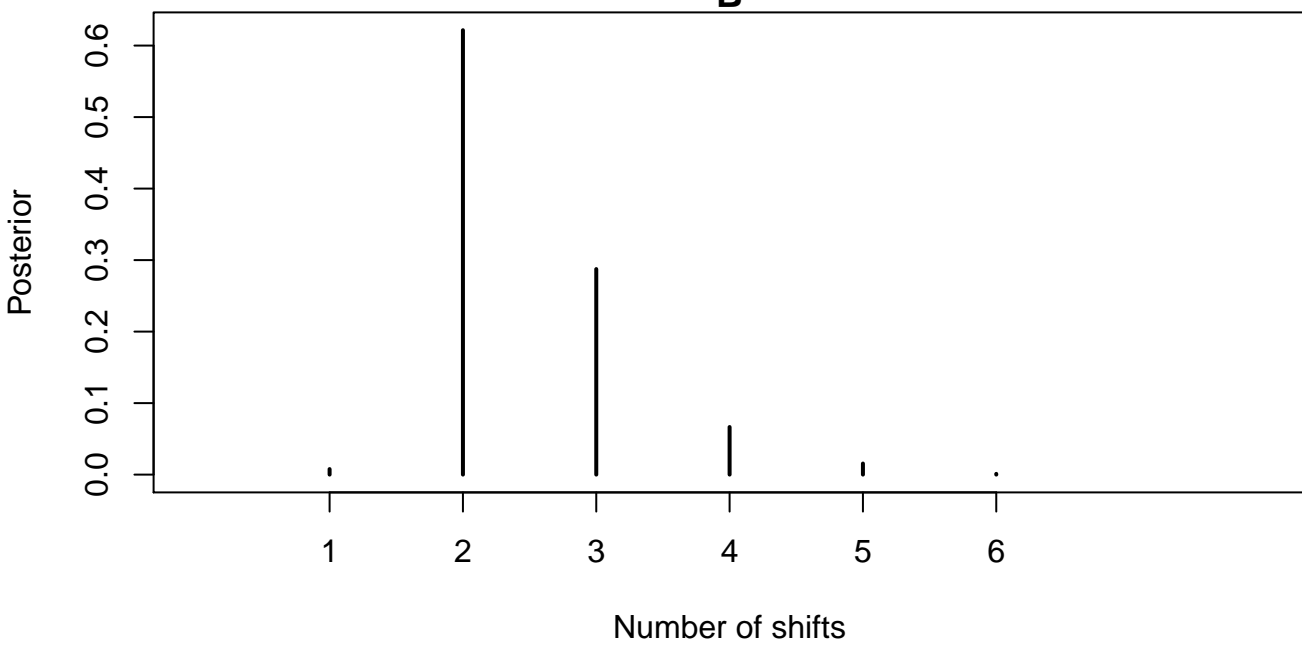

Supplement: Additional file 5: Figure S4. — Frequency distribution of distinct macroevolutionary rate regimes estimated using BAMM and the tree reconstructed with the Yule prior. (A) Prior distribution of the number of distinct processes. (B) Posterior distribution of the number of distinct processes (including the root process) on the cycad phylogeny reconstructed with a Yule model. A one-process model outperforms a two-process model. [file 12862_2015_347_MOESM5_ESM.pdf]

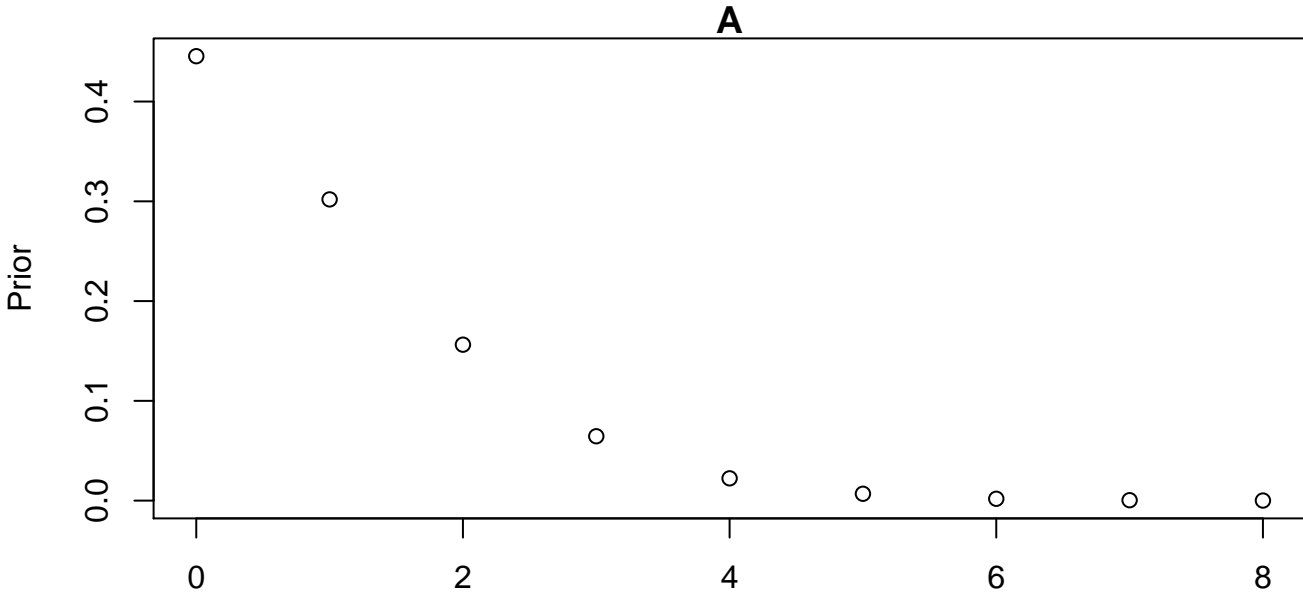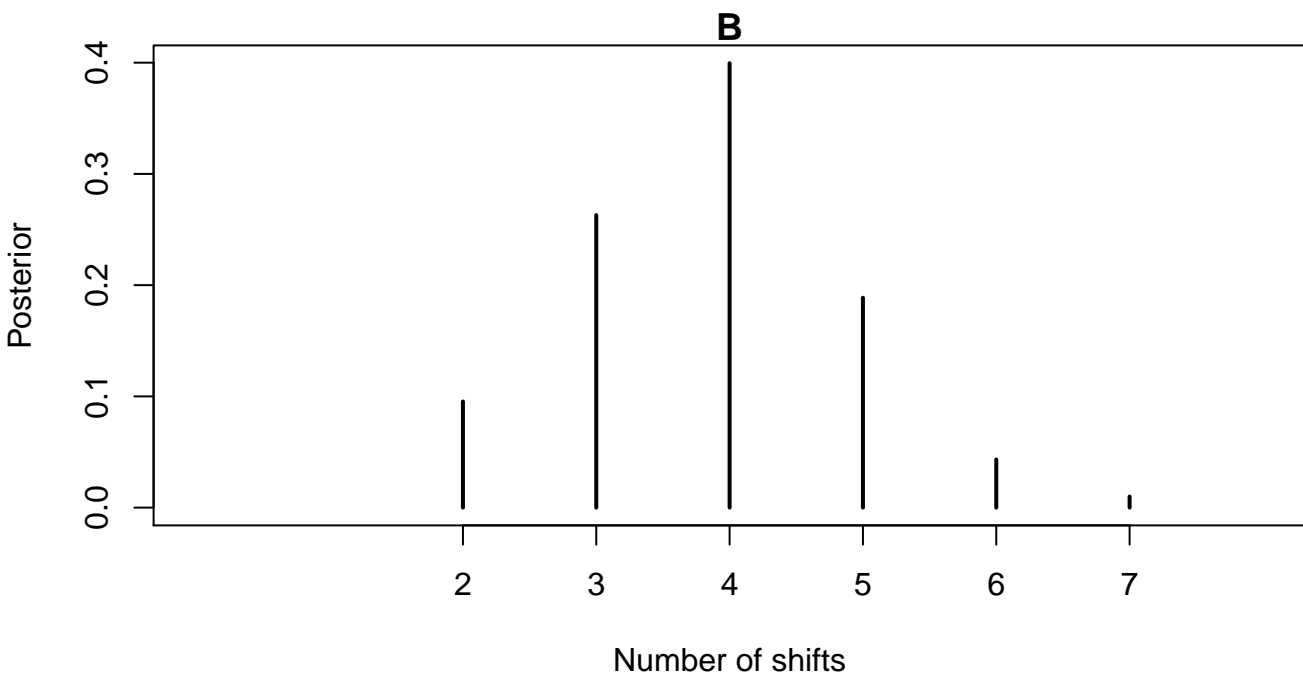

Supplement: Additional file 6: Figure S5. — Frequency distribution of distinct macroevolutionary rate regimes estimated using BAMM and the tree reconstructed with the birth-death prior. (A) Prior distribution of the number of distinct processes. (B) Posterior distribution of the number of distinct processes (including the root process) on the cycad phylogeny reconstructed with a birth-death model. A one-process model outperforms a two-process model. [file 12862_2015_347_MOESM6_ESM.pdf]

A

 $f = 0.33$ 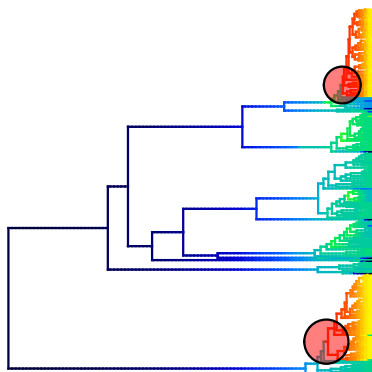 $f = 0.11$ 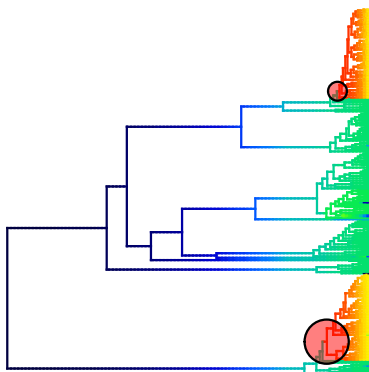 $f = 0.092$ 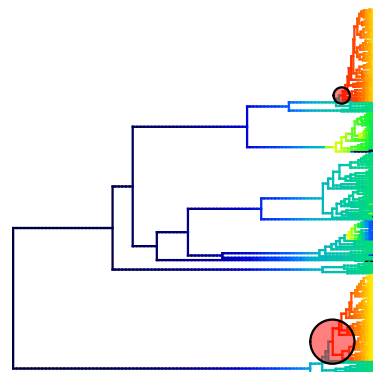 $f = 0.073$ 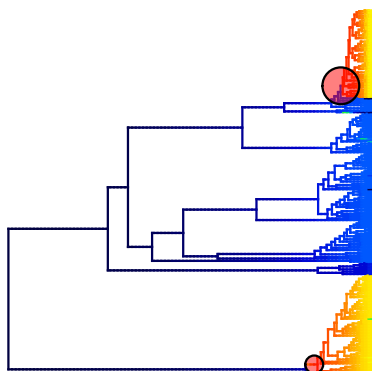 $f = 0.032$ 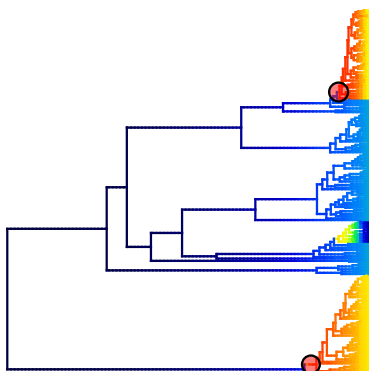 $f = 0.027$ 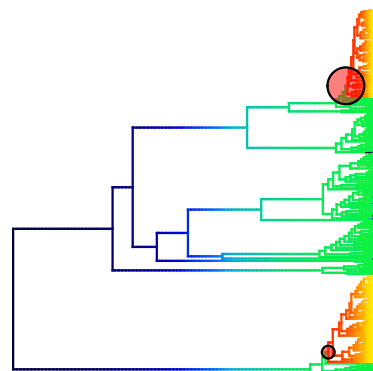 $f = 0.024$ 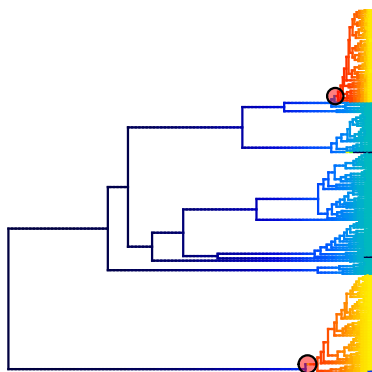 $f = 0.024$ 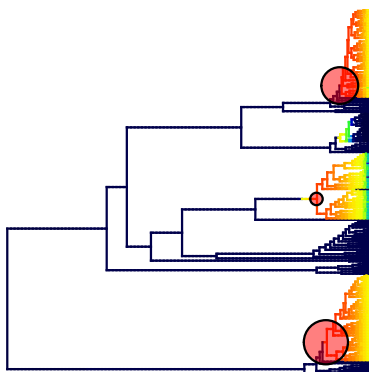 $f = 0.018$ 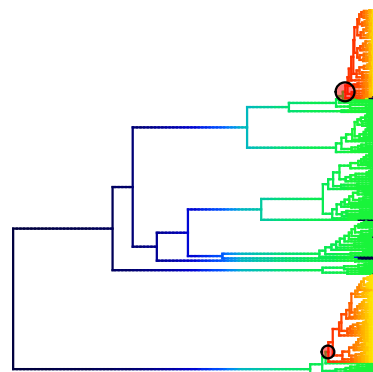

B

$$f = 0.067$$
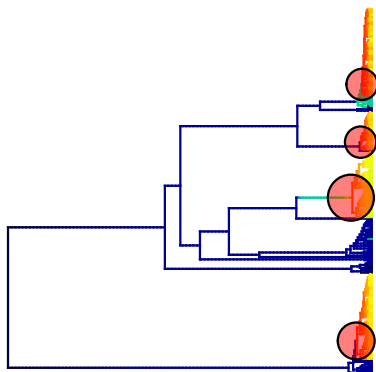
$$f = 0.042$$
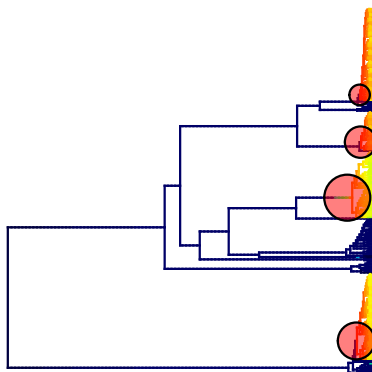
$$f = 0.038$$
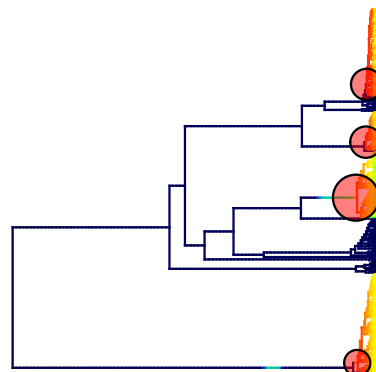
$$f = 0.032$$
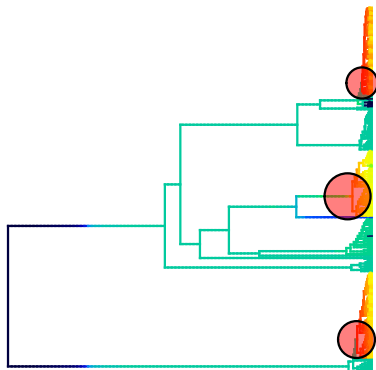
$$f = 0.032$$
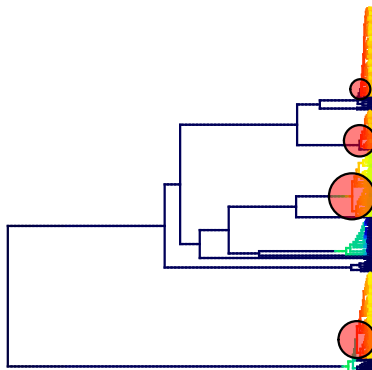
$$f = 0.032$$
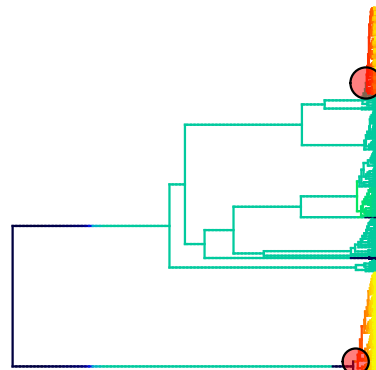
$$f = 0.029$$
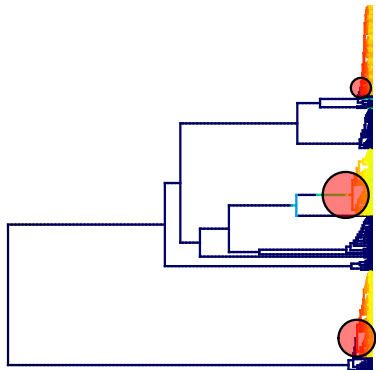
$$f = 0.027$$
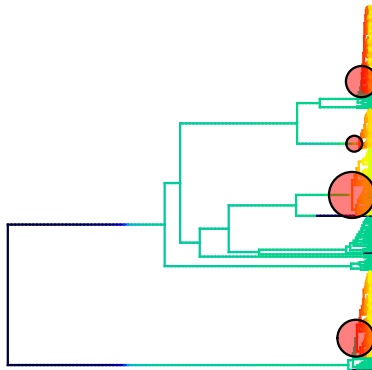
$$f = 0.027$$
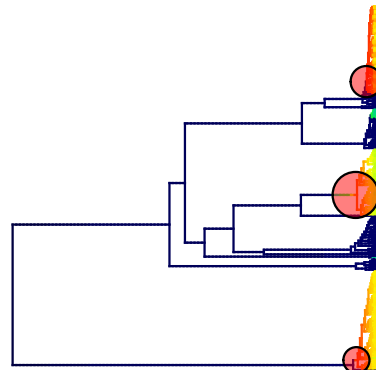

Supplement: Additional file 7: Figure S6. — Credible set of configuration shifts of cycads inferred with BAMM using the (A) tree with the Yule prior, and the (B) tree dated with the birth-death prior. Phylogenies show the distinct shift configurations with the highest posterior probability. For each shift configuration, the locations of rate shifts are shown as red (rate increases) and blue (rate decreases) circles, with circle size proportional to the marginal probability of the shift. Text labels (e.g. f =0.33) denote the posterior probability of each shift configuration. [file 12862_2015_347_MOESM7_ESM.pdf]

A

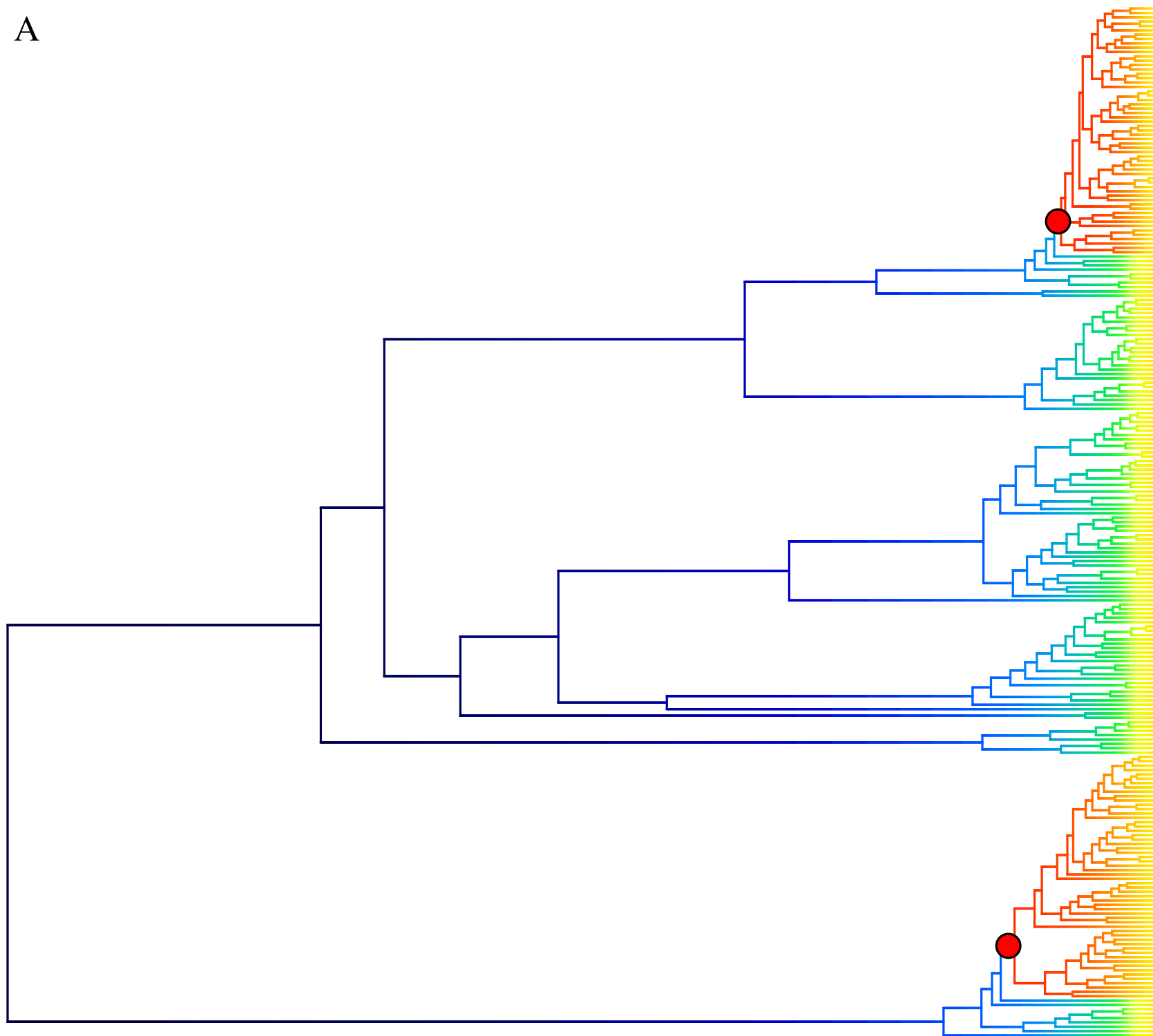

B

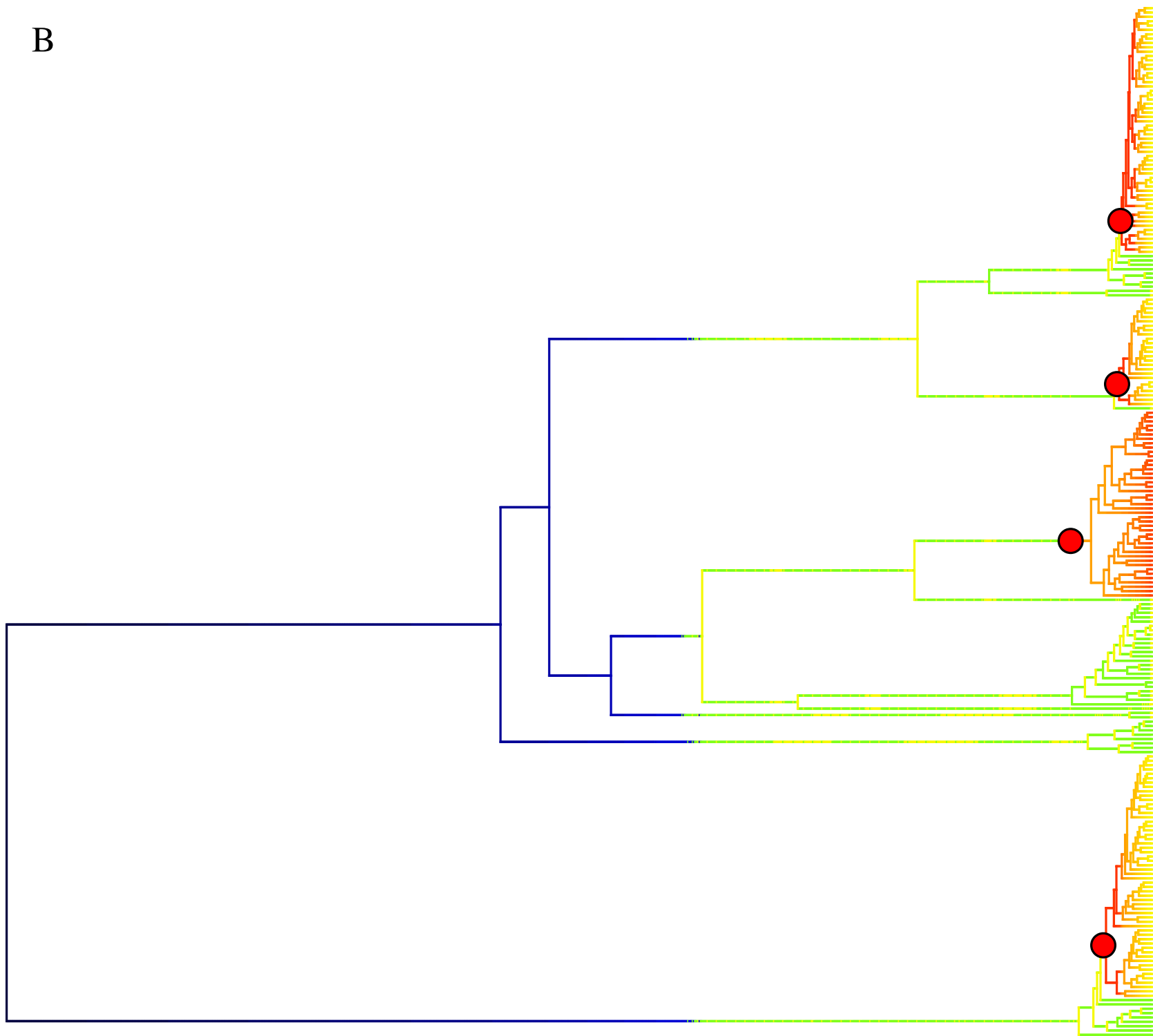

Supplement: Additional file 8: Figure S7. — The best shift configuration inferred with BAMM using the (A) tree with the Yule prior, and the (B) tree dated with the birth-death prior. Analysis with the Yule prior indicated a single rate shift (indicated by red circle) near the crowns of Cycas and Encephalartos, whereas the analysis with the birth-death prior indicated four major shifts at the richest genera: Cycas, Zamia, Encephalartos, and Macrozamia. [file 12862_2015_347_MOESM8_ESM.pdf]

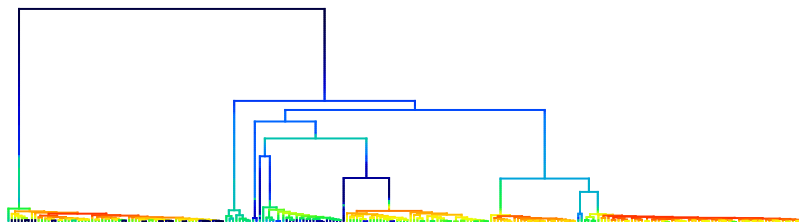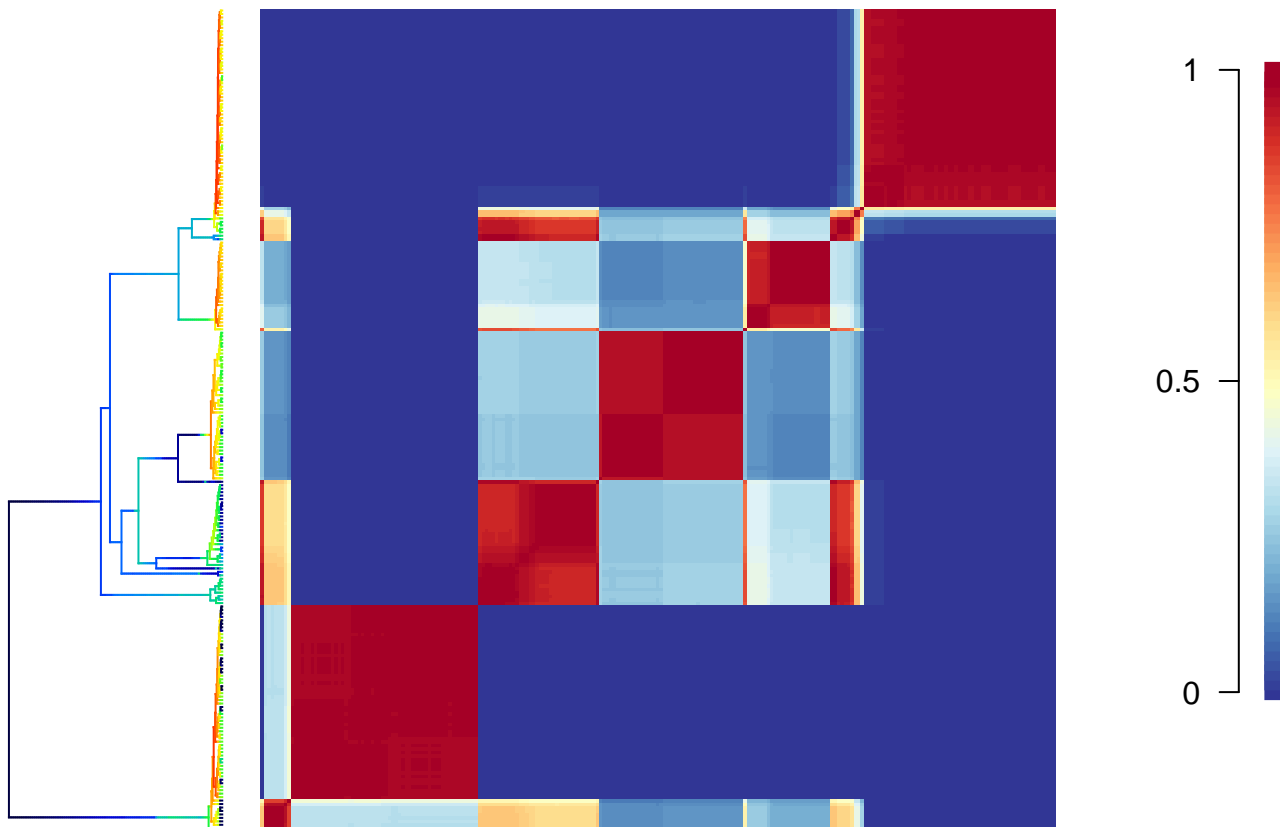

Supplement: Additional file 9: Figure S8. — Macroevolutionary cohort matrix for speciation of cycads using the birth-death prior. Each cell in the matrix is coded by a color denoting the pairwise probability that two species share a common macroevolutionary rate regime. The cycad phylogeny reconstructed with a birth-death process is shown for reference on the left and upper margins of each cohort matrix. At least five major cohorts can be identified that are the four most species-rich genera: Cycas, Zamia, Encephalartos, and Macrozamia. [file 12862_2015_347_MOESM9_ESM.pdf]

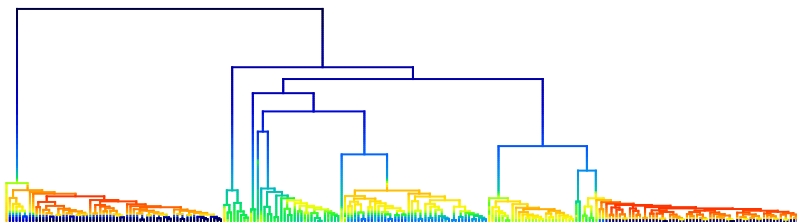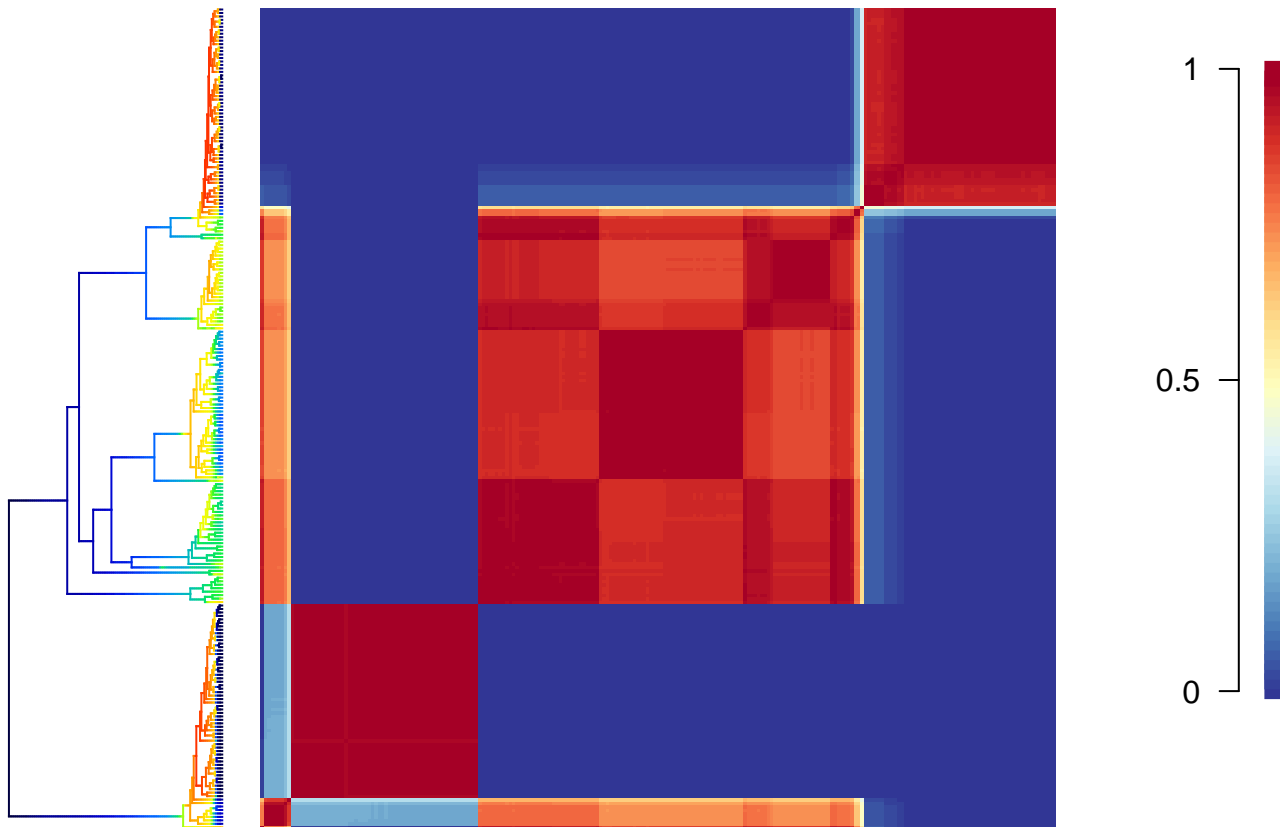

Supplement: Additional file 10: Figure S9. — Macroevolutionary cohort matrix for speciation of cycads using the Yule prior. Same legend as Additional file 9: Figure S8. [file 12862_2015_347_MOESM10_ESM.pdf]
